# Worsening drought of Nile basin under shift in atmospheric circulation, stronger ENSO and Indian Ocean dipole

**DOI:** 10.1038/s41598-022-12008-8

**Published:** 2022-05-16

**Authors:** Shereif H. Mahmoud, Thian Yew Gan, Richard P. Allan, Jianfeng Li, Chris Funk

**Affiliations:** 1 Department of Civil and Environmental Engineering, University of Alberta, Edmonton, T6G 1H9 Canada; 2 Department of Meteorology, University of Reading, Berkshire, RG6 6BB UK; 3 Department of Geography, Hong Kong Baptist University, Hong Kong, China; 4 Climate Hazards Center, University of California Santa Barbara, Santa Barbara, CA 93106 USA

**Keywords:** Climate sciences, Hydrology, Engineering

## Abstract

Until now, driving mechanisms behind recurring droughts and hydroclimate variations that controls the Nile River Basin (NRB) remains poorly understood. Our results show significant hydroclimatic changes that contributed to recent increasing aridity of NRB since the 1970s. Besides climate warming, the influence of stronger ENSO and Indian Ocean dipole (IOD) in NRB has increased after 1980s, which have significantly contributed to NRB’s drought severity at inter-annual to inter-decadal timescales. Our results demonstrate that warming, El Niño and IOD have played a crucial role on NRB’s inter-decadal hydroclimate variability, but IOD has played a more important role in modulating NRB’s hydroclimate at higher timescales than El Niño. Results also indicate that the impacts of positive phases of ENSO and IOD events are larger than the negative phases in the NRB hydroclimate. Further, the southward (westward) shift in stream functions and meridional (zonal) winds caused an enhancement in the blocking pattern, with strong anticyclonic waves of dry air that keeps moving into NRB, has resulted in drier NRB, given stream function, geopotential height and U-wind anomalies associated with El Niño shows that changes in regional atmospheric circulations during more persistent and stronger El Niño has resulted in drier NRB. After 1970s, El Niño, IOD, and drought indices shows significant anti-phase relationships, which again demonstrates that more frequent and severe El Niño and IOD in recent years has led to more severe droughts in NRB. Our results also demonstrate that IOD and and the western pole of the Indian Ocean Dipole (WIO) are better predictors of the Nile flow than El Niño, where its flow has decreased by 13.7 (upstream) and by 114.1 m^3^/s/decade (downstream) after 1964. In summary, under the combined impact of warming and stronger IOD and El Niño, future droughts of the NRB will worsen.

## Introduction

Since the beginning of ancient civilizations in Africa, the Nile River has been the major source of water supply to its eleven riparian countries. However, since the 1970s, recurring droughts^1–4^, changes to the timing and amount of precipitation, and increasing population have led to rising tension between competing users for water and growing political instability^5^. The Fifth Assessment Report (AR5) of the Intergovernmental Panel on Climate Change (IPCC) concluded that drying had occurred over much of Africa and the number of hydrologic extremes and heat stress have doubled since the middle of the twentieth century^5,6^. Past studies predominantly confined to a specific sub-basin and have not provided us with a clear perspective on the driving mechanisms behind recurring droughts and the hydroclimate variations that controls the Nile River Basin (NRB). Among various causes of droughts identified in Africa^1–4^ are the decline in precipitation related to warming caused by rising concentration of greenhouse gases^4,7^. Past studies also show that precipitation in the Blue Nile Basin (BNB) ^8–11^ with high spatial and temporal variability is affected by El Niño Southern Oscillation (ENSO) such that positive anomalies (wet years) tend to occur during the negative phase of ENSO ^12–14^, while negative anomalies (dry years) during the positive phase of ENSO. Additionally, the BNB’s precipitation and flow tend to be high during La Niña years but low during El Niño years^15–17^, and extreme droughts correspond to strong El Niño events. The frequency of severe droughts occurring in NRB at inter-annual to inter-decadal time scales is linked to a long period of below average precipitation^3^. An important question to address is: are NRB droughts primarily caused by precipitation anomalies attributed to ENSO/IOD, or have other factors also contributed to its drought severity, such as the warming trend of Africa^18^, increasing frequency of extreme El Niño events^16,19^, and changes in atmospheric circulation^20^ associated with anthropogenic greenhouse gas or aerosol forcing, volcanic effects or internal unforced variability^21^. In view of limited knowledge and lack of detailed analysis of the hydroclimatic changes in the NRB, there is an urgent need to better understand changes in the hydrological cycle of NRB.

**Figure 1:**
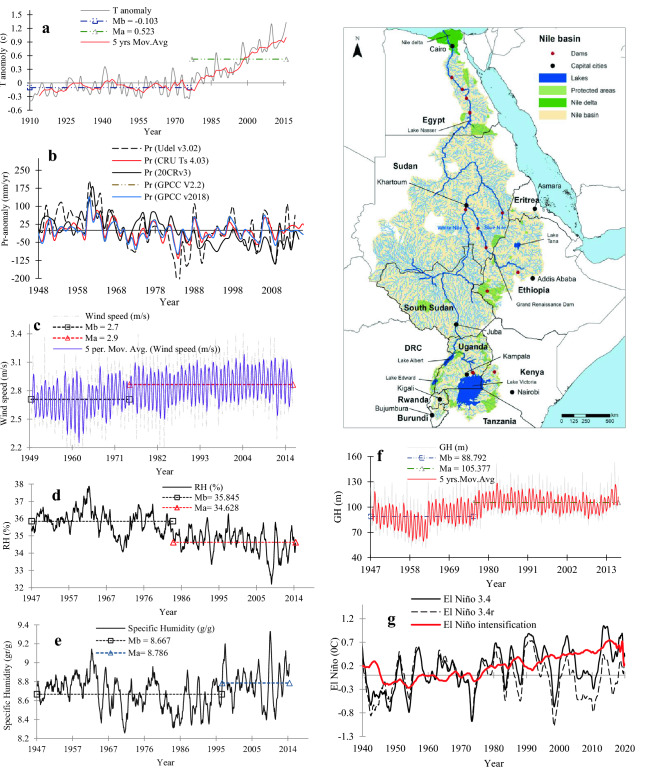
A detailed map of the Nile River Basin (NRB) basin showing the main riparian of the Nile generated with ArcMap Version 10.1 (http://www.esri.com/en/arcgis/arcgis-for-desktop/). The observed streamflows analysed are taken from the Blue Nile station in Khartoum, Dongola station, and the Aswan station. The analysed hydroclimate data and drought index are averaged over the NRB, as well as for each riparian country of the Nile. Figure 1 shows: (**a**) temperature anomaly, where Mb is the long-term average before the change point, and Ma the long-term average after the change point. (**b**) precipitation anomaly from five different datasets, (**c**) wind speed, (**d**) relative humidity, (**e**) specific humidity, (**f**) geopotential height, and (**g**) ERSST relative (without global warming signal) and absolute El Niño3.4 index time series, Fig. 1g also shows El Niño3.4 intensification by global warming (red-line). Figure 1 also shows a decreasing trend in precipitation at 16.2 mm/decade since 1970s, increasing trend in wind speed and zonal wind stress at 0.02 m/decade and 1.51 m^2^/s^2^/decade respectively since 1975, increasing trend in geopotential height (GPH) at 3.1 m/decade since 1976, warming trend at 0.19 °C/decade, and decreasing trend in relative humidity (RH) since 1977.

The flow of NRB comes from two sources, the Blue Nile, and the White Nile, which join at Khartoum, the capital city of Sudan (Fig. 1). The BNB occupies about 11% of the NRB but it contributes about 60% of the Nile River flow^22^. With a total catchment area of 3.1 million km^2^, the NRB is shared by eleven countries, namely, Burundi, Rwanda, Uganda, Kenya, Tanzania, South Sudan, Democratic Republic of Congo, Sudan, Eritrea, Ethiopia, and Egypt. About 86% of NRB’s area lies in Sudan, Ethiopia, and Egypt. Despite its recurrent occurrences, we have yet to explain droughts of NRB satisfactorily because most past drought studies mainly focus on precipitation anomalies, soil moisture, and vegetation indices of the BNB^8–10,12^. Here, we analyzed the hydroclimate data of NRB to identify key driving forces behind climate warming and droughts in NRB, and the variability of the Nile flow data over 1900–2012 in three gaging stations, and implications of flow variability to the severity and intensity of hydrological droughts in each riparian country of the NRB. We also identify mechanisms that control sensitivities of droughts, and the teleconnection of droughts and hydroclimate of NRB to ENSO and the dipole modes of the Indian Ocean. This study is first to investigate the full range of possible climate change impacts on NRB’s hydroclimate, droughts, and correct some results reported in past studies. The results from this study would help us to develop more effective mitigation strategies for these riparian countries against the potential impact of future droughts.

## Results and discussion

### Abrupt changes in the NRB’s hydroclimate under climate warming

To conduct a broad-scale analysis of the hydroclimatic changes of NRB, Pettitt's test and Mann–Kendall (MK) were applied to monthly precipitation, surface temperature, geopotential height, relative humidity, specific humidity, potential and actual evapotranspiration, wind speed, zonal and meridional wind stresses, Nile flow, surface runoff, soil moisture content (SMC), and total water storage (TWS) data of the NRB (see methods sections). The NRB has experienced significant climate change impact in recent decades, as shown in a statistically significant change point (1976) in surface temperature, with a statistically significant warming trend of 0.19 °C/decade over 1976–2017 (Fig. 1a, supplementary Table 1). A statistically significant change point was also detected (1970) in the monthly precipitation anomaly data from five different datasets (Udel., GPCC, 20CRv3, CRU.TS4.03, and GPCC V2018) with an overall decreasing trend of 16.2 mm/decade (Fig. 1b) and increasing trend in wind speed and zonal wind stress (NCEP– NCAR) at 0.02 m/decade and 1.51 m^2^/s^2^/decade respectively since 1975 (Fig. 1c). For the 1948–2017 relative humidity data (RH) (20CRv3), a statistically significant change point was detected in 1977, with a significant decreasing trend of 0.35% /decade after 1977 (Fig. 1d). Similarly, a statistically significant change point (1976) was detected in monthly 1000-mb geopotential height (GPH) data (20CRv3), and an increasing trend of 3.1 m/decade (Fig. 1f). As expected, higher surface temperature had resulted in higher GPH^23^, which means the lower atmosphere had become warmer. A statistically significant change point (1994) was also detected in the monthly specific humidity data of 1948–2017, and a slight increasing trend of 0.15 g/kg per decade, with the mean specific humidity increasing from 8.6 g/g in 1948–1994 to 8.8 g/g in 1994–2017 (Fig. 1e).

In addition, from the 1950–2017 reference and actual evapotranspiration (AET) estimated for NRB using a surface energy balance algorithm (see methods section), the AET of NRB has increased significantly with an upward trend of 14.4 mm/decade (supplementary Fig. 1b, and supplementary Table 2) since 1990s. Furthermore, a statistically significant change point was detected in monthly SMC and TWS in 1979, and after the change point, SMC and TWS data exhibit significant negative trends of 0.84 mm/decade and 1.44 mm/decade, respectively (supplementary Figs. 2c and 3c). Analysis of Niño3.4 data shows a statistically significant change point in 1978 (Fig. 1g), and statistically significant increasing trend of 0.17 °C/decade. Since 1970s, changes between the El Niño index and the “relative” El Niño^24^ (relative to 20S-20 N, i.e., without global warming trend) shows that El Niño have become stronger with higher intensity in recent years (Fig. 1g). To further confirm the location of the abrupt variations in the NRB’s hydroclimate, seven commonly used nonparametric single and multiple change detection methods were applied to the NRB’s hydroclimate data (see methods section). Supplementary Table 2 shows the exact location of these variations using each detection method. The results obtained from these methods confirm the location of the hydroclimate variations in the NRB. For instance, posterior probability-based methods sch as BCP, PELT, and Pettitt clearly confirm the statistically significant change points in NRB’s precipitation, RH, wind speed, specific humidity, GPH, AET, SMC, and TWS in 1970, 1977, 1975, 1994, 1976, 1995, and 1979, respectively.

**Figure 2:**
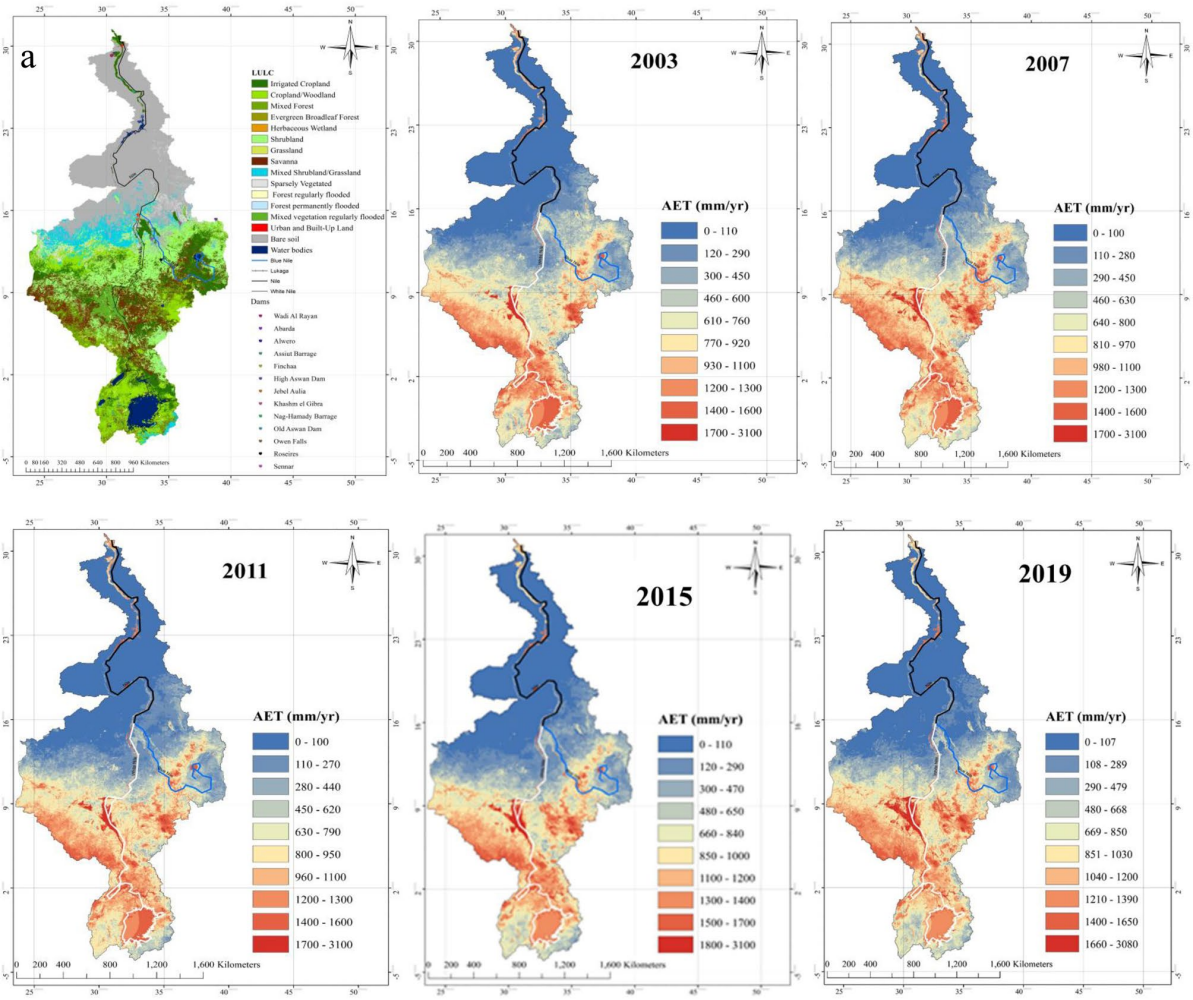
Spatial distribution of annual reference evapotranspiration (**a**), land cover (**b**), and AET (2003–2019). In (**a**) the average annual reference evapotranspiration for the NRB ranged from 746 to 2340 mm/year, with the highest values observed over Ethiopian highlands and the southern portion of the NRB in countries such as Uganda, Egypt, Sudan, Burundi, Congo, Kenya, Rwanda, and Tanzania. Our results also show that the NRB’s AET rates follow the rainy seasonal pattern. In (**b**) very high AET is observed in irrigated land and water bodies in these regions which lead to very high losses due to increased rate of AET. Figure 2 also shows that AET increases from year to year, this increasing trend expand to include other regions in the NRB. The maps were generated with ArcMap Version 10.1 (http://www.esri.com/en/arcgis/arcgis-for-desktop/).

A composite analysis of NRB’s hydroclimate data between 1948 and 2017 also shows significant changes across the entire NRB (supplementary Figs. 4 and 5). For instance, surface temperature has increased at 0.16–0.4 °C/decade over the NRB, with the highest increase in Ethiopia, Uganda, Sudan, and Egypt (supplementary Fig. 4a). RH has also decreased by 1–5%/decade after 1985, with the largest decrease in Ethiopia, Uganda, and Sudan where warming has also been the worst (supplementary Fig. 4b). The SMC shows high spatial variabilities in NRB but at a decreasing trend of 16–45 mm/decade between 1985 and 2017 (supplementary Fig. 4c). The lower atmosphere stream function of supplementary Fig. 4f at 0.8458 sigma level, which depicts the rotational part of the flow (the flow is along the contours), indicates that main waves emanating from northern towards southern parts of NRB, have shifted further south from 1948–1984 to 1985–2017. These changes had resulted in lower daily precipitation (supplementary Fig. 5a), marginally higher specific humidity (supplementary Fig. 5b), lower annual surface runoff (supplementary Fig. 4d), but higher PET (supplementary Fig. 4e). At a positive trend of 0.2–0.8 m^2^/sec^2^ between 1985 and 2017, the increase in scalar wind was maximum in Uganda, Sudan, and northwestern regions of Ethiopia, where both meridional and zonal wind have also increased (supplementary Fig. 5c–e). These results also show that higher wind speed and wind stresses tend to blow away humid air from land, resulting in a drier atmosphere. This long-term southward shift in the stream function over NRB would have also contributed towards the long-term drying of NRB, as part of multiple changes attributed to climate warming, e.g., changes in precipitation, Ts, wind stresses, GPH, RH, SMC, TWS, surface runoff, and AET.As expected, higher AET is found in irrigated land and water bodies in the Ethiopian highlands and in countries of southern NRB such as Uganda, Egypt, Sudan, Burundi, Congo, Kenya, Rwanda, and Tanzania, where water losses from high AET can be substantial (Fig. 2).

### Attribution of changes in NRB’s hydroclimate

**Figure 3:**
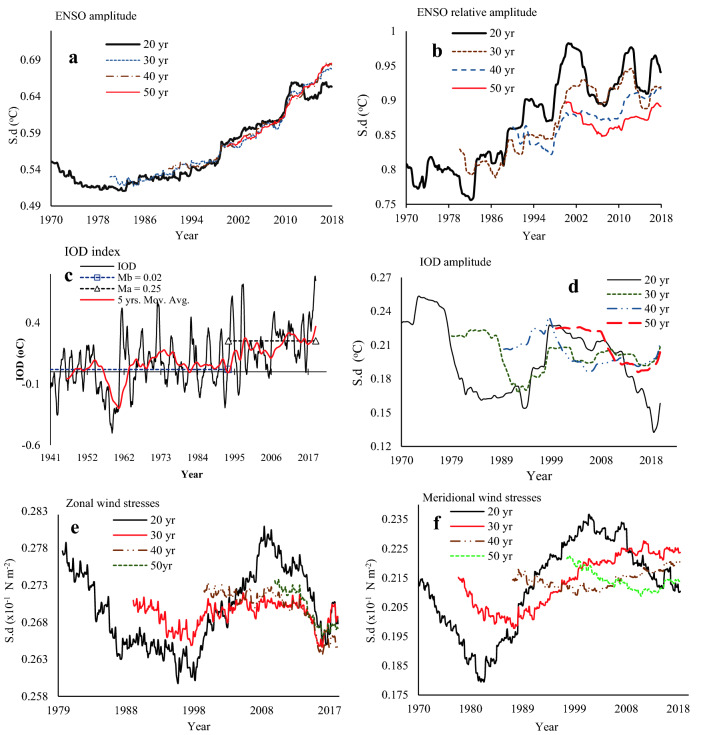
Observed ENSO and Indian Ocean dipole (IOD) amplitude, and zonal and meridional winds stresses over the NRB: ENSO and IOD amplitude (°C), defined as the standard deviation (s.d.) of the Niño3.4 and relative El Niño3.4 (El Niño3.4r) amplitude (**a**,**b**) and IOD index (**d**) over 20-, 30-, 40- and 50-year windows from 1950 to 2017, and IOD characteristics over the NRB (**c**) using the ERSST data sets. Zonal and meridional wind stresses amplitude (10^−1^ N m^−2^) are defined as the s.d. of zonal and meridional winds stresses over 20-, 30-, 40- and 50-year windows from 1950 to 2017, using NCEP/NCAR data sets from 1950–2017 (**e**,**f**). An increasing meridional wind amplitude (**e**) associated with a prominent anticyclonic circulation in southern NRB have contributed to the observed increasing intensity of recent El Niño events (**a**,**b**).

To examine how ENSO and IOD amplitudes vary over at inter-annual to inter-decadal timescales and their influence on the observed warming and changes in atmospheric circulation over the NRB, we computed their amplitudes over 20-, 30-, 40- and 50-year window following the method adopted in Kim et al.^16^. From the SD (standard deviation) of the Niño3.4 index and relative El Niño3.4 (El Niño3.4r) estimated over 20-, 30-, 40- and 50-year windows in Fig. 3a,b, it is clear that the degree of ENSO variability has increased in NRB over the 1980–2017 period with increasing intensity in ENSO activities signified by several extreme El Niño events occurring over this period. The difference between El Niño amplitudes with and without the global warming signal in Fig. 3a,b clearly shows that ENSO events have grown to be stronger in recent years. As a result, the impact of El Niño events in the hydroclimate of NRB have become stronger by climate warming, which have contributed to the observed long-term trends in Fig. 1 since the 1970s. Figure 3b also suggest that natural variability modulates ENSO amplitude over multidecadal time scales^25^, as demonstrated by SD of Niño3.4r over 20-, 30- 40-, and 50-year windows, which consistently show that the amplitude of Niño3.4r variability has enhanced over the past several decades even after removing the global warming signal. In Fig. 3d, the Indian Ocean dipole (IOD) defined by a zonal SST gradient, also exhibited increasing variability similar to ENSO, where the SD of IOD over 1970–2018 was characterized by strong and frequent occurrences of positive events associated with El Niño events. Figure 3c also shows a statistically significant change point in 1993, and increasing trend of about 0.1 °C/decade, for IOD had been in a positive phase since 1993. Figure 3e,f also show that higher zonal wind stresses are associated with stronger El Niño, their amplitude in terms of wind speed and discharging effect also increased with stronger El Niño events , which could have also contributed to more severe aridity in NRB, such as statistically significant increasing trends in the warm spell duration and maximum daily temperature over NRB at about 3.1 day/decade and 0.35 °C/decade since 1975, respectively (supplementary Fig. 1a and supplementary Table 1).

**Figure 4:**
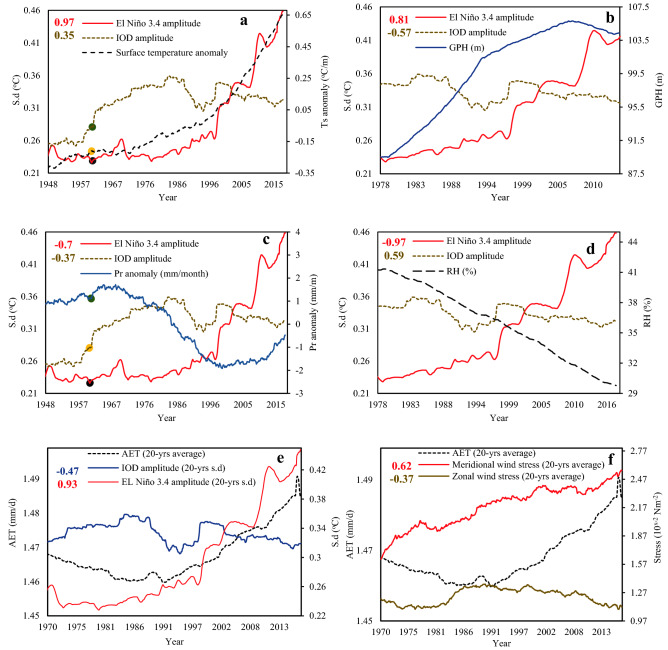
ENSO and IOD Teleconnections on NRB hydroclimate. IOD and El Niño 3.4 amplitudes correlations with NRB surface temperature (**a**), GPH (**b**), precipitation anomaly (**c**), relative humidity (**d**), AET (**e**), and AET correlation with meridional and zonal wind stress (**f**). Surface temperature anomaly, GPH, precipitation anomaly, and relative humidity are computed over 20-year running periods from 1920 to 2017, El Niño 3.4 and IOD amplitude are the SD of El Niño 3.4 and IOD indexes over 20-year windows from 1920 to 2017 using the ERSST data sets. The numbers in the top right are the cross-correlation coefficient between variables and IOD amplitude (brown colour) and El Niño 3.4 amplitude (red colour) at the 5% level. In (**a**,**c**) black and yellow points represent neutral ENSO conditions and strong positive IOD in 1961, green point is the response on surface temperature “increase” and decrease in precipitation anomalies “decrease” in the same year.

Our analysis also shows that the influence of stronger El Niño and IOD in NRB has increased after 1970s, particularly the influence of IOD on NRB’s hydroclimate over inter-decadal timescales. The IOD’s power spectrum and significant coherence with ENSO, demonstrate a strong coupling between them, for both exhibited similar change patterns, e.g., positive IOD becomes more intensive as the strength of El Niño increases (supplementary Fig. 6). As a result, the impact of El Niño and IOD events in the hydroclimate of NRB have increased because of climate warming, which have contributed to the observed long-term trends in NRB’s hydroclimate since the 1970s. A wavelet coherence analysis (WTC) shows that the hydroclimate of NRB is strongly correlated with El Niño and IOD (supplementary Figs. 7 and 8). The strong WTC (anti-phase) between El Niño and IOD, and precipitation and RH after 1970s shows that El Niño and IOD had contributed to lower precipitation (drying) over NRB, and their significant in-phase relationship with surface temperature and GPH shows that stronger El Niño (El Niño3.4r) and IOD after 1970s resulted in a warmer NRB. In addition, WTC plots between AET and El Niño show in-phase, statistically significant coherent relationship at 2–4 and 8–14-years bands after 1970s (supplementary Fig. 9a, b), which peaked at 14–16-year time scale after 2000s. On the other hand, the WTC between IOD and AET show that IOD primarily lead AET after 2000s. Based on WTC and a detailed detrended cross correlation (ρ) analysis (DCCA), the hydroclimate of NRB is strongly teleconnected to El Niño and IOD (Fig. 4) at inter-annual to inter-decadal timescales, with positive or negative correlations between El Niño 3.4, surface temperature (ρ = 0.97) (Fig. 4a), GPH (ρ = 0.81) (Fig. 4b), RH (ρ = − 0.97) (Fig. 4c) and precipitation (ρ = − 0.7) (Fig. 4d).

The strong correlation between these climate variables and IOD show that IOD plays an important role on the hydroclimate variability of NRB. For instance, the strong negative correlation between IOD, precipitation (ρ = − 0.37) (*p*-value < 0.05) and the Standardized Precipitation Index (SPI) (*ρ* = − 0.87) demonstrate that besides El Niño, IOD has also contributed to lower precipitation and more severe droughts of NRB (Figs. 4d and 6a–f), similar to the teleconnection between East African precipitation, ENSO and IOD^26^. The higher AET after 1970s can be partly attributed to stronger wind stresses associated with stronger El Niño amplitudes, as shown by strong WTC between zonal and meridional wind stresses and AET at 1–2-year bands (Fig. 4e–f, supplementary Figs. 9 and 10). DCCA also showed a significant positive correlation between AET and El Niño 3.4 (ρ = 0.93), and meridional wind stresses (ρ = 0.62). Apparently, increasing meridional wind stress anomalies and stronger El Niño and IOD events have both contributed to the increased aridity in NRB (Fig. 4f, supplementary Fig. 10). It is shown that whenever drought occurs, the air temperature tends to be higher than average because more net solar energy is received (less clouds) while less energy is used to evaporate water or soil moisture^27^.

**Figure 5:**
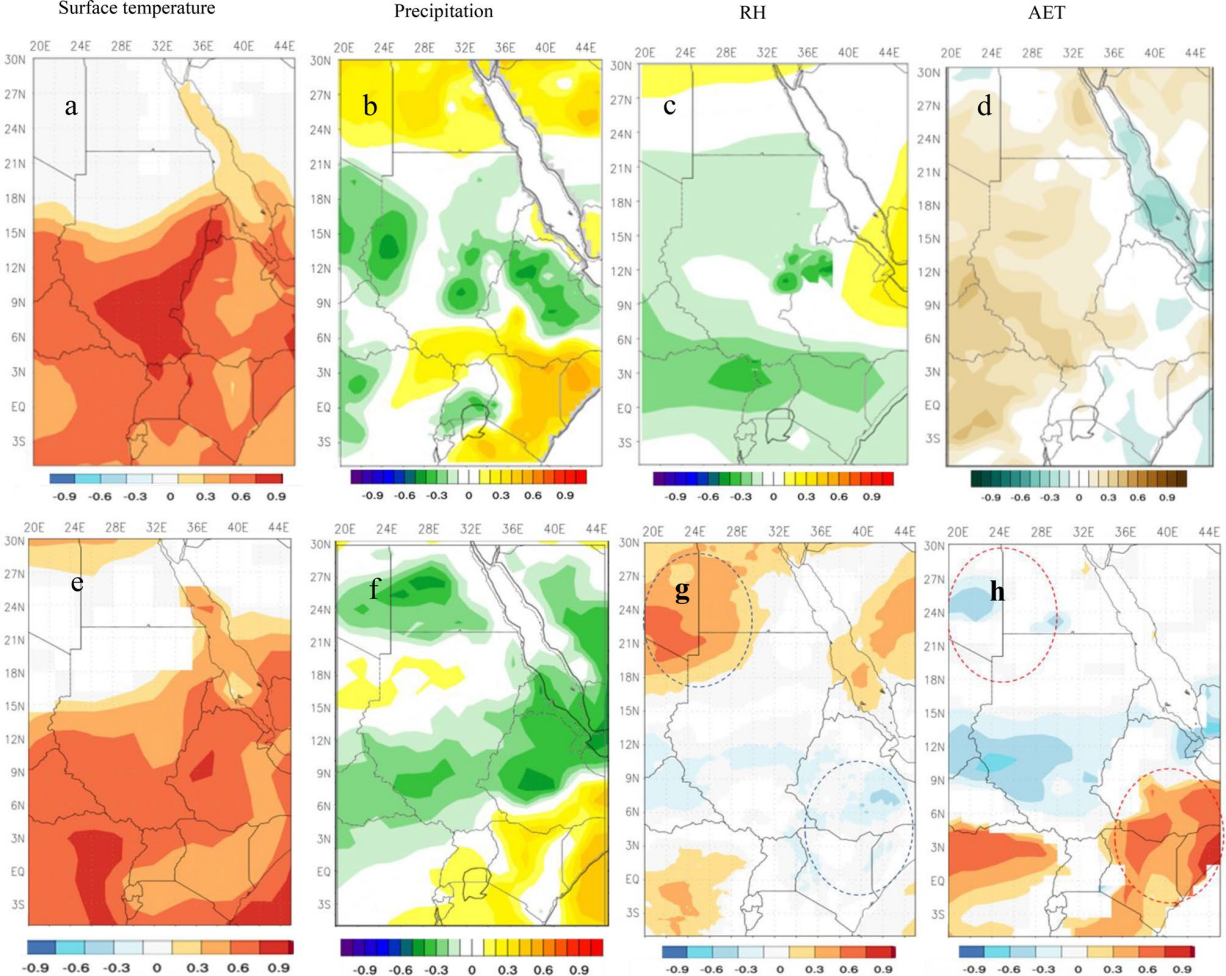
Spatial correlation between NRB’ surface temperature, precipitation, relative humidity, and AET with El Niño 3.4 (**a**–**d**) and IOD (**e**–**h**) between 1948:2017 (*p* < 5%). In (**b**) the strong negative correlation between El Niño and precipitation in lowland of Ethiopia, Sudan, Uganda, Rwanda, and Burundi indicates that El Niño plays a dominant role in precipitation variability (reduction) in these countries. On the other hand, there is a strong positive correlation between El Niño and precipitation in Kenya, Tanzania, and Egypt implying an increase in precipitation in these countries. In (**d**) the spatial relationship between AET and El Niño is a mirror-opposite of the relative humidity—El Niño relationship (**c**) because of the strong dependence between relative humidity and evaporation rate i.e., a decrease in relative humidity causes evaporation rate to increase. In e, IOD intensified ENSO-induced warming in the upper part of the NRB. In addition, IOD seems to amplify ENSO impact on the NRB’s precipitation variability with reduced precipitation in Eritrea, Ethiopia, Sudan, Congo, and Egypt, and increased precipitation over Uganda, Kenya, Tanzania, Burundi, and Rwanda (**f**). In g and h, RH and AET show an almost opposite pattern to their relationship with ENSO. The maps were generated with NCAR Command Language (NCL) Version 6.2.1 (http://www.ncl.ucar.edu/).

To better understand the independent influence of El Niño and IOD on each riparian country of NRB, we estimated spatial correlation between IOD and El Niño and NRB’s hydroclimate using the independent composite method adopted in Saji and Yamagata^28^. Our findings show that the influence of El Niño over NRB’s hydroclimate varies widely across the entire basin spatially as shown by the spatial correlation between El Niño and NRB’s GPH, Ts, precipitation, RH, and AET for the past 70 years. El Niño events have had a stronger warming effect in the upper part of the NRB, as shown by the strong positive correlation $(\rho$ = 0.3–0.9) between surface temperature and El Niño in Ethiopia, Kenya, Uganda, Rwanda, Burundi, Tanzania, Eretria, and Sudan (Fig. 5a), leading to higher warm spell duration over NRB at about 3.1 day/decade since 1975 (supplementary Fig. 1a), which is expected given more frequent and severe El Niño events in recent years. These results are within agreement with ENSO-induced warming over Kenya (0.15 °C /decade) and Ethiopia (0.3 °C /decade) since 1970s^29,30^. In addition to El Niño-induced warming in NRB, the spatial correlation between NRB’s precipitation and El Niño shows a strong negative (positive) correlation between El Niño and precipitation in lowland of Ethiopia, Sudan, Uganda, Rwanda, and Burundi (Kenya, Tanzania, and Egypt) (Fig. 5b). This finding agrees with that of de la Poterie et al.^31^, who reported above (below) average precipitation in Kenya (Ethiopia) during El Niño years.

Furthermore, RH (AET) is negatively (positively) correlated with El Niño in the Ethiopian’s lowlands, Sudan, South Sudan, Uganda, Kenya, Eritrea, Burundi, and Rwanda (Fig. 5c,d), which shows that a decrease (increase) in NRB’s RH (AET) attributed to El Niño warming effect in the NRB since the 1970s. Byrne and O’gorman^32^ have also found a similar relationship between land RH and increased warming in recent years. The results of the DCCA, WTC and trend analysis between El Niño and the NRB’s hydroclimate over the past 70 years provide evidence of El Niño induced changes on the NRB’s hydrological cycle. Our results also indicate that El Niño induced impacts on the NRB’s hydroclimate can either be intensified or decreased, depending on the strength of IOD events. For instance, the spatial correlation between NRB’s surface temperature and IOD in Fig. 5e shows a mirror image of El Niño warming signal over the NRB’s riparian countries. We also demonstrate that IOD amplifies the impact of ENSO on NRB’s precipitation variability, as shown by the significant negative correlation between IOD and precipitation in Eritrea, Ethiopia, Sudan, Congo, and Egypt (Fig. 5f). Beside El Niño, IOD has also contributed to lower precipitation in these countries, an opposite pattern to the teleconnection between East African precipitation, ENSO and IOD^26^, which shows positive correlation with ENSO and IOD. Figure 5f also shows a significant positive correlation between IOD and precipitation in Uganda, Kenya, Tanzania, Burundi, and Rwanda. The negative influence of IOD on Ethiopian’s precipitation was also shown by Kotecha and Barnston^28^. In contrast to El Niño induced impacts on the NRB’s RH and AET over the past 70 years, our findings show that IOD have a positive effect on NRB’s RH (increase) i.e., lower AET (Fig. 5g,h). This is evident in the statistically significant positive (negative) correlation between IOD and RH over Egypt and northern parts of Sudan (Ethiopian highlands, Kenya, and Tanzania). In other words, the relationship between NRB’s RH and AET and IOD shows an opposite pattern to their relationship with El Niño. Figure 5g,h also shows that IOD reduced El Niño negative influence on RH over Egypt and northern parts of Sudan and intensified the influence over Ethiopian highlands, Kenya, and Tanzania resulting in higher AET over these countries.

**Figure 6:**
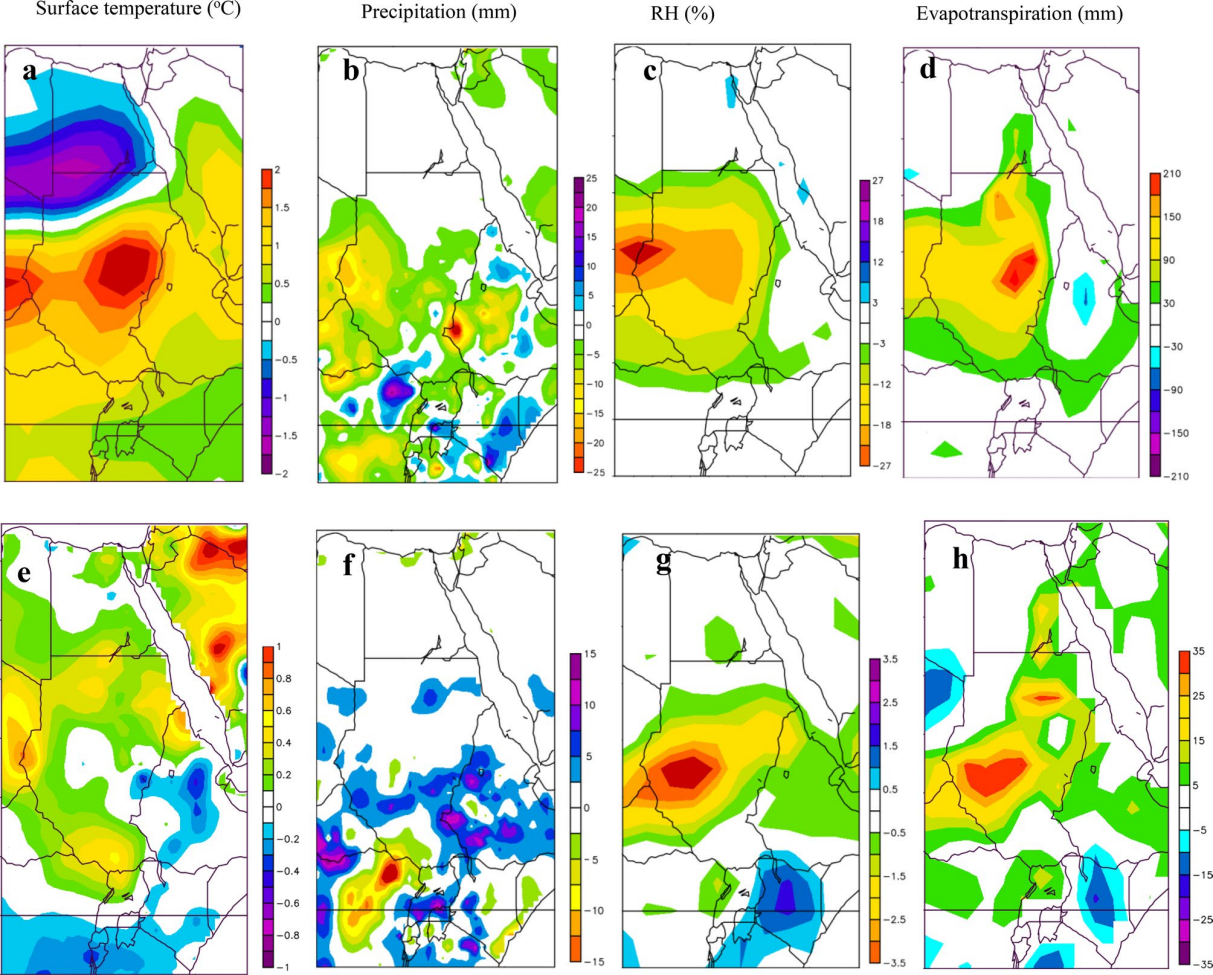
The effect of El Niño and La Niña events in the NRB shown by composite of NRB’ surface temperature associated with El Niño (**a**) and La Niña (**e**) episodes, composite of precipitation associated with El Niño (**b**) and La Niña (**f**) episodes, composite of relative humidity associated with El Niño (**c**) and La Niña (**g**) episodes, and composite of evapotranspiration associated with El Niño (**d**) and La Niña (**h**) episodes.The maps were generated with NCAR Command Language (NCL) Version 6.2.1 (http://www.ncl.ucar.edu/).

We have analyzed composites of separate phases of ENSO and IOD to identify the distinct features of each phase in in NRB annual surface temperature, precipitation, RH, and evapotranspiration. First, pure El Niño and La Niña events were identified and were compared to non-ENSO/IOD events following the approach adopted in Meyers et al.^33^. Then, NRB surface temperature, precipitation, RH, and evapotranspiration during each phase were averaged and subtracted from the average of the nonevents to form the composite. The same approach was followed to form the composite of positive and negative phases of IOD. Differences between El Niño and La Niña composites show that the impacts of El Niño are larger than La Niño in the NRB (Fig. 6). For instance, the surface temperature anomaly composites of pure El Niño events (Fig. 6a) show higher than normal surface temperature over the upper part of the NRB (0.5–2 °C), and lower than normal downstream (mainly over Egypt). The precipitation anomaly composites of pure El Niño events in Fig. 6b show below average precipitation over the upper part of the NRB (− 5 to − 25 mm). The highest increase (decrease) in surface temperature (precipitation) anomaly during pure El Niño events can be seen over lowland of Ethiopia, Sudan, Uganda, Rwanda, and Burundi. During pure El Niño events, the upper part of the NRB experiences a large decrease in RH (− 3 to − 27%) (Fig. 6c), resulting in higher-than-normal evapotranspiration (30–210 mm) (Fig. 6d). Overall, we note that evapotranspiration is the highest above countries that showed a large increase (decrease) in surface temperature (RH). Despite the cooling influence of La Niña events, surface temperature is warmer than normal in the majority of the NRB (0.2 to 1 °C) except for Ethiopia which shows a drop of − 0.2 to − 0.4 °C (Fig. 6e). In contrast, above normal precipitation occurs in most parts of the NRB during pure La Niña events (5–15 mm). On the other hand, Fig. 6g–h show slight decrease (increase) in RH (evapotranspiration) in comparison to the pattern during pure El Niño events.

**Figure 7:**
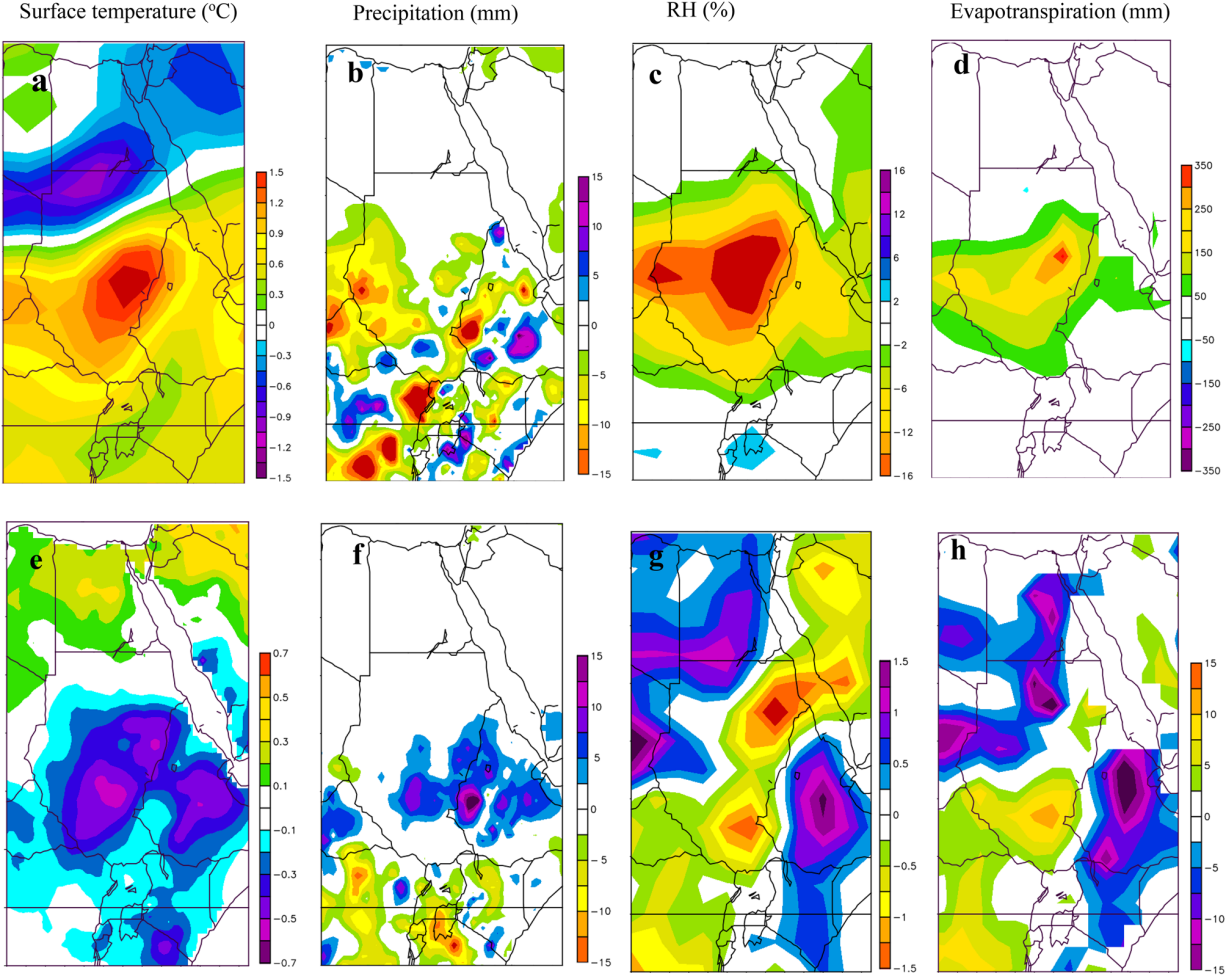
The effect of positive and negative IOD events in the NRB shown by composite of NRB’ surface temperature associated with positive IOD (**a**) and negative IOD (**e**) episodes, composite of precipitation associated with positive IOD (**b**) and negative IOD (**f**) episodes, composite of relative humidity associated with positive IOD (**c**) and negative IOD (**g**) episodes, and composite of evapotranspiration associated with positive IOD (**d**) and negative IOD (**h**) episodes. The maps were generated with NCAR Command Language (NCL) Version 6.2.1 (http://www.ncl.ucar.edu/).

Figure 7 shows a far stronger influence from the positive phase of IOD than the negative phase IOD in the NRB’ climate. For instance, surface temperature anomaly composites of positive IOD events shows higher than normal surface temperature over the upper part of the NRB (0.3–1.5 °C) (Fig. 7a), this is a mirror image of the pure El Niño warming signal, but the influence is slightly lower. The composites of precipitation in Fig. 7b show below normal precipitation over the upper part of the NRB (− 3 to − 15 mm), which indicates that positive IOD events intensify the influence of positive ENSO events in the NRB precipitations. Like positive ENSO events, positive IOD events caused a decrease in RH and increase in evapotranspiration over the upper part of the NRB (Fig. 7c,d). On the other hand, negative IOD events impose a widespread colder-than-average surface temperature over the upper part of the NRB (− 0.1 to − 0.7 oC), and wormer than average surface temperature over Egypt (0.1–0.7 °C) (Fig. 7e). Negative IOD events also associated with higher-than-average precipitation (5–15 mm) over Ethiopia and Sudan, and below average precipitation (− 5 to − 15 mm) over Kenya, Uganda, Tanzania, Rwanda, and Burundi (Fig. 7f). Figure 7g–h also shows increase (decrease) in RH (evapotranspiration) over Ethiopia, Kenya, and Tanzania. In contrast, to lower (higher) than average RH (evapotranspiration) over Sudan. Overall, we conclude that the impacts of positive phases of ENSO and IOD events are larger than the negative phases in the NRB hydroclimate.

### The influence of ENSO and IOD on drought conditions of NRB

Past studies have teleconnected droughts of the upper part of the NRB^9,10^ and sub-Saharan Africa^21^ to ENSO based on a single drought index such as SPI or Normalized vegetation index (NDVI). However, in our study, the frequency, intensity, change point and trend of the meteorological, agricultural, and hydrological droughts of NRB under the effect of climate change were investigated based on the SPI index, the Normalized vegetation index (NDVI), the Standardized Precipitation-Evapotranspiration Index (SPEI), the self-calibrating Palmer Hydrological Drought Index (sc-PDSI), and runoff anomaly, estimated over 1950–2017, respectively (see methods section). In addition, to further investigate drought conditions and hydroclimate of the NRB, sc-PDSI, surface temperature, and precipitation of each riparian country of NRB were analyzed individually (supplementary Table 3). Even though the degree of climate warming in NRB varies from one riparian country to another, overall impacts to meteorological, agricultural, and hydrological droughts of most riparian countries of NRB have been severe, where regional warming trends have exceeded the mean global warming trend of about 0.15 °C per decade^34^.

**Figure 8:**
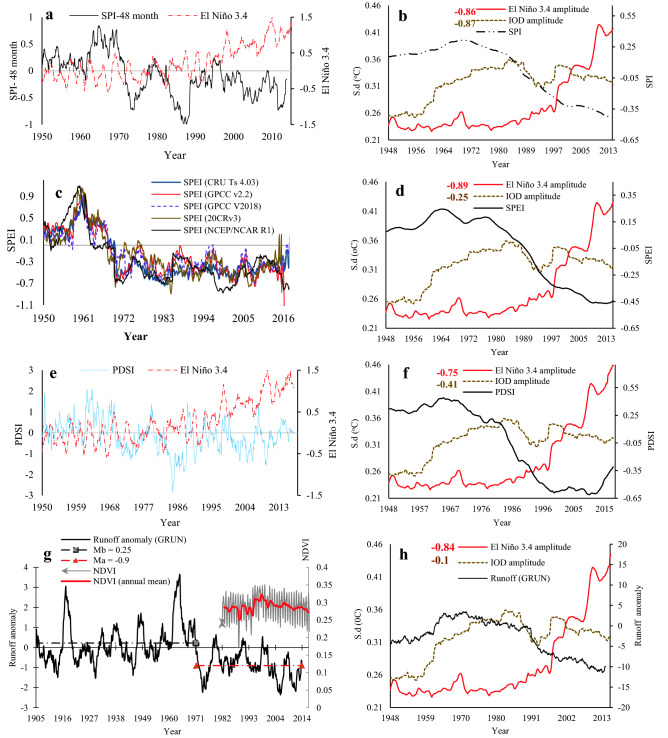
The influence of ENSO and IOD on drought conditions of NRB: (**a**) 48-month SPI versus El Niño 3.4, (**b**) IOD and El Niño 3.4 amplitudes correlations with SPI, (**c**) SPEI calculated using five precipitation data sets, all SPEI indices show a statistically significant drying trend (*p* < 0.05) since the 1970s. (**d**) IOD and El Niño 3.4 amplitudes correlations with SPEI, (**e**) sc-PDSI versus El Niño 3.4, (**f**) IOD and El Niño 3.4 amplitudes correlations with sc-PDSI, (**g**) trend and variability of monthly runoff anomaly averaged over a 3-year moving window between 1902 and 2014 using GRUN- Runoff observation-based global gridded runoff. The right-hand side of Fig. 8g shows the monthly Normalized Difference Vegetation Index (NDVI) computed from NOAA Climate Data Record (CDR) of AVHRR NDVI V5 and averaged over the entire Nile River basin. (**h**) IOD and El Niño 3.4 amplitudes correlations with runoff anomaly. In (**b**,**d**,**f**,**h**) the SPI, SPEI, sc-PDSI, runoff indices were computed over 20-year running periods from 1920 to 2017, El Niño 3.4 and IOD amplitude are the SD of El Niño 3.4 and IOD indexes over 20-year windows from 1920 to 2017 using the ERSST data sets. The numbers in the top right of (**b**,** d**,** f**, and** h**) are the detrended cross-correlation coefficient between drought indices and IOD amplitude (brown colour) and El Niño 3.4 amplitude (red colour) at the 5% level.

Beside ENSO, our results show that IOD plays a more crucial role on NRB’s hydroclimate variability and drought severity over inter-decadal and longer timescales (Fig. 8 and supplementary Fig. 8). The SPI index was computed at 1, 3, 6, 12 and 48-month timescales for NRB over 1950–2017. The 1-month SPI time series shows statistically significant change point in 1979 with a decreasing trend of 0.15/decade (supplementary Table 4). By defining droughts as SPI < − 1, recurrent droughts were detected in 1952, 1959, 1965, 1972, 1973, 1978, 1983, 1984, 1987, 1991, 1994, 1999, 2002, and 2011, respectively, with increasing severity after late 1970s. The 48-month SPI (Fig. 8a) also exhibits overall decreasing trends since 1970s, but of higher magnitude than the 1-month SPI. To consider the effect of warming and potential evapotranspiration (PET) on drought severity, the SPEI index at 1, 3, 6, 12 and 48-month timescale for 1940–2018 was computed over the NRB from five precipitation datasets (Udel., GPCC, 20CRv3, CRU.TS4.03, and GPCC V2018). Like the SPI index, the 48-month SPEI index from the five datasets consistently shows a statistically significant change point in 1983 with a decreasing trend of 0.1–0.15/decade (Fig. 8c). The consistent results obtained from the above five datasets confirm that higher PET intensified by warming had a significant impact on NRB during the most dominant drought events between 1970 and 2018. The sc-PDSI time series for NRB also shows a significant change point in 1983, with an overall decreasing trend of 0.58/decade, as reflected by hydrologic droughts in 1973 and 1987, and then recurrent droughts between 2002 and 2011 (Fig. 8e). This means that more frequent hydrologic droughts have occurred over NRB than the global average since early 2000s^35^.

In NRB, rain-fed agriculture is the prevailing agricultural system, and thus negative runoff anomalies are good indicators for hydrologic droughts as well as lower agricultural productivity. The monthly observed runoff anomalies in NRB between 1902 and 2014 in Fig. 8g show a statistically significant change point in 1970 with a decreasing trend of 1.2 mm/decade. Figure 8g also shows the response (drying) of agricultural and vegetation cover (right-hand side of Fig. 8g) to the decline in runoff between 1981 and 2014. The negative monthly runoff anomaly after 1970s indicate a lower runoff availability to rain-fed agriculture and ecosystems and increases the severity of droughts in NRB. Before 1970s, the WTC plots show that El Niño and IOD were in phase with both sc-PDSI, SPI, and SPEI, but after 1970s their relationships became predominantly anti-phase. These posts 1970s results suggest that stronger El Niño and IOD events contributed to more severe droughts in NRB after 1970s (Fig. 8c–e and supplementary Fig. 8e–f), as is also evident from the strong negative correlation between El Niño (IOD) and SPI (Fig. 8b), ρ = − 0.86 (− 0.87), SPEI (Fig. 8d), ρ = − 0.89 (− 0.25), sc-PDSI (Fig. 8f), ρ = − 0.75 (− 0.41), and runoff anomaly (Fig. 8h), ρ = − 0.84 (− 0.39). Results obtained from using NDVI shows that an increase in AET and a decline in precipitation could lead to severe agricultural droughts (supplementary Fig. 11) in irrigated areas of the Ethiopian highlands, Ethiopia, Eritrea, Kenya, Tanzania, Congo, and Uganda since the 1970s.

The role of the changes in NRB’s hydroclimate, hydrological cycle and warming-induced drought stress is evident in the consistent results obtained from drought indices representing meteorological, agricultural, and hydrological droughts in NRB, which show repetitive drought episodes, with increasing severity after 1970s. These indices support the archived historical information on drought events in NRB. For instance, the 48-month SPI exhibits overall decreasing trends since 1970s, the SPEI index computed over the NRB from five precipitation datasets confirm that higher AET intensified by warming had a significant impact on NRB during the most severe drought events that occurred 1970 and 2018, and the sc-PDSI index also reflected recurrent hydrologic droughts in the NRB after 1970s. These findings show that more frequent hydrologic droughts have occurred over NRB than the global average since early 2000s^35^, resulting in severe agricultural droughts in Ethiopia, Eritrea, Kenya, Tanzania, Congo, and Uganda since the 1970s.

### The role of western and southeastern poles of the Indian Ocean Dipole on NRB’s hydroclimate and drought

To further investigate the role of IOD on the hydroclimate of NRB, using the two halves of IOD identified in Saji et al.^36^, i.e., the western pole in the Arabian Sea (western pole of the Indian Ocean Dipole) (50° E–70° E, 10° S–10° N) (WIO) and the southeast pole of the Indian Ocean (SEIO) (90° E–110° E, 10° S–0° N) we found that SST of the WIO plays a primary role on NRB’s hydroclimate over inter-decadal and longer timescales. The dominant anti-phase relationship between WIO and NRB’s precipitation, SPI and scPDSI at inter-decadal timescales (> 32-year) show that NRB’s hydroclimate is strongly linked to the SST of WIO (supplementary Fig. 12a–f), which is also evident in the significant negative correlation between WIO and the precipitation variability of NRB (ρ = − 0.82). Apparently, more frequent occurrences of droughts in NRB are related to increased warming in the WIO (supplementary Fig. 13a) and other factors, as is also evident from the strong negative correlation between SPI and SST of WIO (ρ = − 0.71 for SPI and − 0.8 for sc-PDSI) (supplementary Fig. 13b–c). There are strong anti-phase relationships at 16–32 (32–64) year bands between WIO (SEIO) and SMC (TWS) (supplementary Fig. 14a–d), with significant negative correlations between NRB’s SMC and SEIO (ρ = -0.92), and WIO (ρ = -0.83) (supplementary Fig. 14e), e.g., NRB’s SMC decreased under stronger SEIO and WIO amplitudes. The strong negative correlation between TWS and SEIO (ρ = − 0.95) and WIO (ρ = − 0.91) implies that increased SST over WIO and SEIO resulted in lower TWS in NRB (supplementary Fig. 14f). Apparently, WIO and SEIO explain the variability of NRB’s SMC and TWS (see supplementary Fig. 14–15). Furthermore, from WTC and strong negative correlations between SPI, sc-PDSI and WIO, NRB’s hydroclimate is shown to be strongly influenced by WIO.

### Responses of Nile flow variability to ENSO and IOD

**Figure 9:**
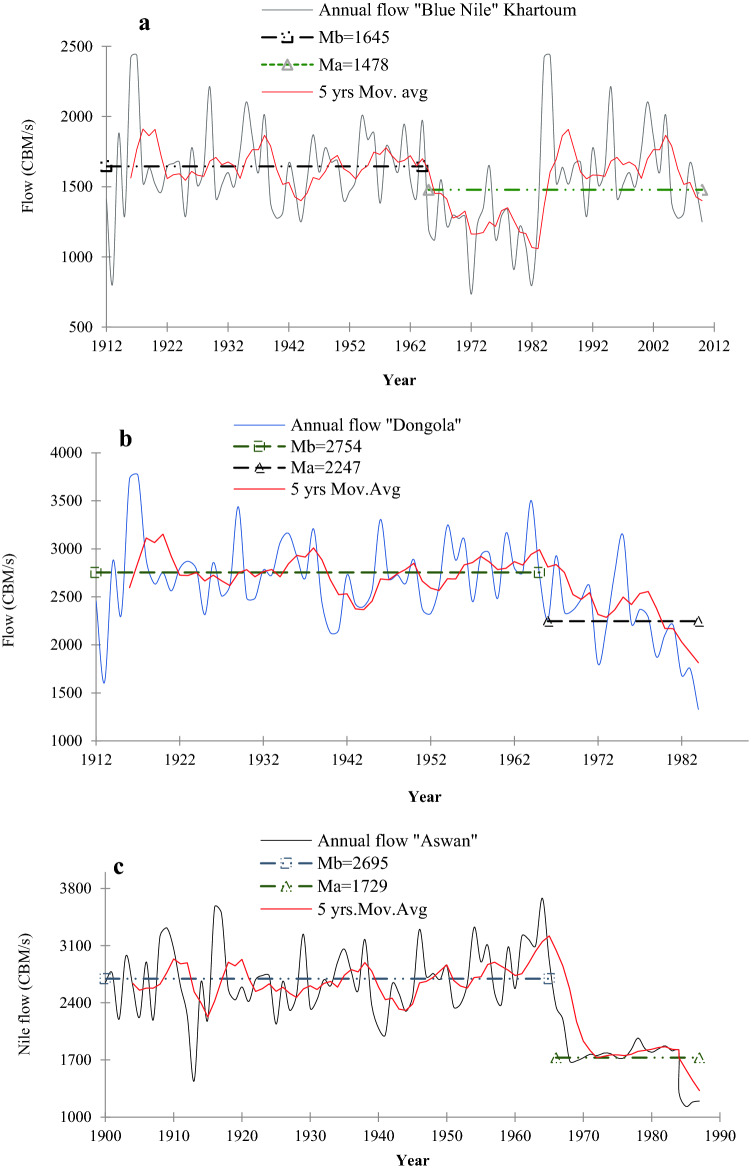
Observed annual flows: (**a**) Blue Nile flow, (**b**) Dongola station flow, and Aswan station flow (**c**). In Fig. 9a, the annual flow of the Blue Nile River decreased from 1645 CBM/s over 1912–1964 to 1478 CBM/s over 1964–2012, with a decreasing trend of about 13.7 CBM/decade after 1964, due to higher AET losses, warmer WIO and IOD, and more intensive El Niño events.

**Figure 10:**
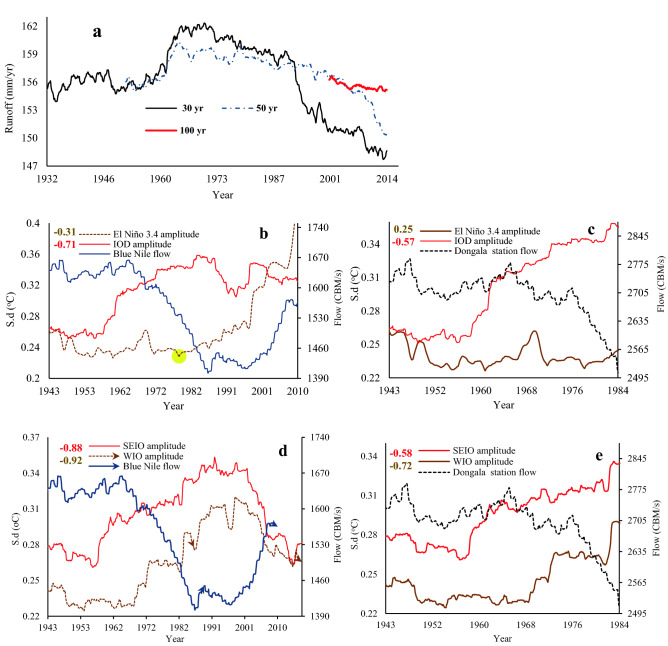
Teleconnections between NRB’s flow variability, ENSO and IOD. (**a**) 30-, 50- and 100-year moving average of annual runoff over the Nile River basin (**a**). Responses of NRB flow variability to El Niño events, IOD amplitude and the IOD two pole amplitudes i.e., western pole amplitude (WIO) and eastern pole amplitude (SEIO) based on the detrended cross correlation between NRB flow at Blue Nile and Dongala stations and El Niño 3.4, IOD amplitudes (**b**,**c**), SEIO, and WIO amplitudes (**d**,**e**), respectively. The NRB flow are computed over 30-year running periods from 1913 to 2012 for the Blue Nile station, and from 1913 to 1984 for Dongala station. El Niño 3.4, IOD, SEIO, WIO amplitudes are computed as the s.d. of the El Niño 3.4, IOD, SEIO, WIO over 30-year windows from 1913 to 2017, using the ERSST data sets.

To better understand hydrologic droughts of NRB, we also investigated the Nile flow variability and the teleconnection of ENSO and the dipole mode to Nile flow over 1912–2012. Observed annual flow records for 1912–2012 from the Blue Nile station at Khartoum (Fig. 9a) show a statistically significant change point in 1964, with a decreasing trend of about 13.7 m^3^/s/decade after 1964. Between 1965 and 1987, the Blue Nile flow decreased so much that the mean annual flow after 1965 was below the long-term mean annual flow by 716 m^3^/s (supplementary Table 5). The annual flow of the Dongola station also showed a significant decline during 1900–1982 (Fig. 9b), while the annual flow of the Aswan station in 1900–1987 exhibited high temporal variability, e.g., the August-November wet season over 1900–1950 shows high flow records, a statistically significant change point in 1965, and a statistically significant negative trend of 114.1 m^3^/s/decade after 1965 (Fig. 9c). The 30-, 50- and 100-yearr moving average of NRB’ annual runoff further indicate lower runoff after 1970s (Fig. 9a). Figure 10a also shows that surface runoff persistently decreases over time as shown by the moving averages. The 30-year moving average of NRB’ annual runoff shows a clear fluctuation, which is attributed to the interannual to decadal oscillation of the NRB’ climate. For instance, the significant increasing trend between 1930 and 1970s indicates higher precipitation, and weaker ENSO, IOD and WIO, as proven by their amplitudes and trends. After the 1970s, we clearly see the shift in the trend from upward to downward. This downward trend in surface runoff accelerated after the 1980s, this interesting finding illustrate the increased influence of IOD, ENSO, and WIO in the NRB’s surface runoff after the 1980s. This pattern is further proven by the longer time scales (i.e., 50- and 100-year moving average) and reflects drought conditions in the NRB at inter-decadal to multi-decadal timescale.

Figure 10b–c shows a significant negative correlation between the Nile flow and IOD (s.d.) (ρ = − 0.71 for the Blue Nile, and − 0.57 for the Nile at Dongala station), compared to a less significant negative/positive correlation between El Niño (s.d.) and the Nile flow at the Blue Nile (ρ = − 0.31) and Dongala stations (ρ = 0.25), respectively. Therefore, IOD exerts a stronger influence on the Nile flow than El Niño at inter-annual to inter-decadal time scales. The peak correlation between IOD and the Nile flow occurred a year earlier than that between El Niño and the Blue Nile flow, which agrees with WTC between IOD and El Niño. The increasing intensity in El Niño (s.d.) after 1978 (represented by the change point (yellow color) in Fig. 10b) shows that El Niño occurred more frequently with positive IODs than La Niña events with negative IODs. Our results show a strong negative correlation between the Nile flow (Blue Nile flow) and the SST of WIO and SEIO (Fig. 10d–e), e.g., between the Nile flow (Blue Nile flow) and WIO, ρ = − 0.72 (− 0.92); and SEIO, ρ = − 0.58 (− 0.88). This demonstrates that the Nile flow is strongly linked to SST of WIO and SEIO (see arrows in Fig. 10d), or to SST of WIO and SEIO, and El Niño at inter-annual to multi-decadal time scales. Contrary to past findings^11,20,22,37^, our results show that IOD and WIO are better predictors of the Nile flow than El Niño. For instance, Siam and Eltahir^37^ provided empirical evidence of the relationship between interannual variability of Nile flow and ENSO without accounting to the influence of IOD in their study. The number of studies that associate the hydroclimate variability of the NRB to IOD and ENSO are very limited and if found these studies focus mainly on the upper part of the NRB. Lastly, given the inter-annual variability of the Nile flow is also projected to increase significantly from the 20th to the twenty-first century^37^, it could lead to even more severe droughts in NRB in future.

### Atmospheric circulation patterns

**Figure 11:**
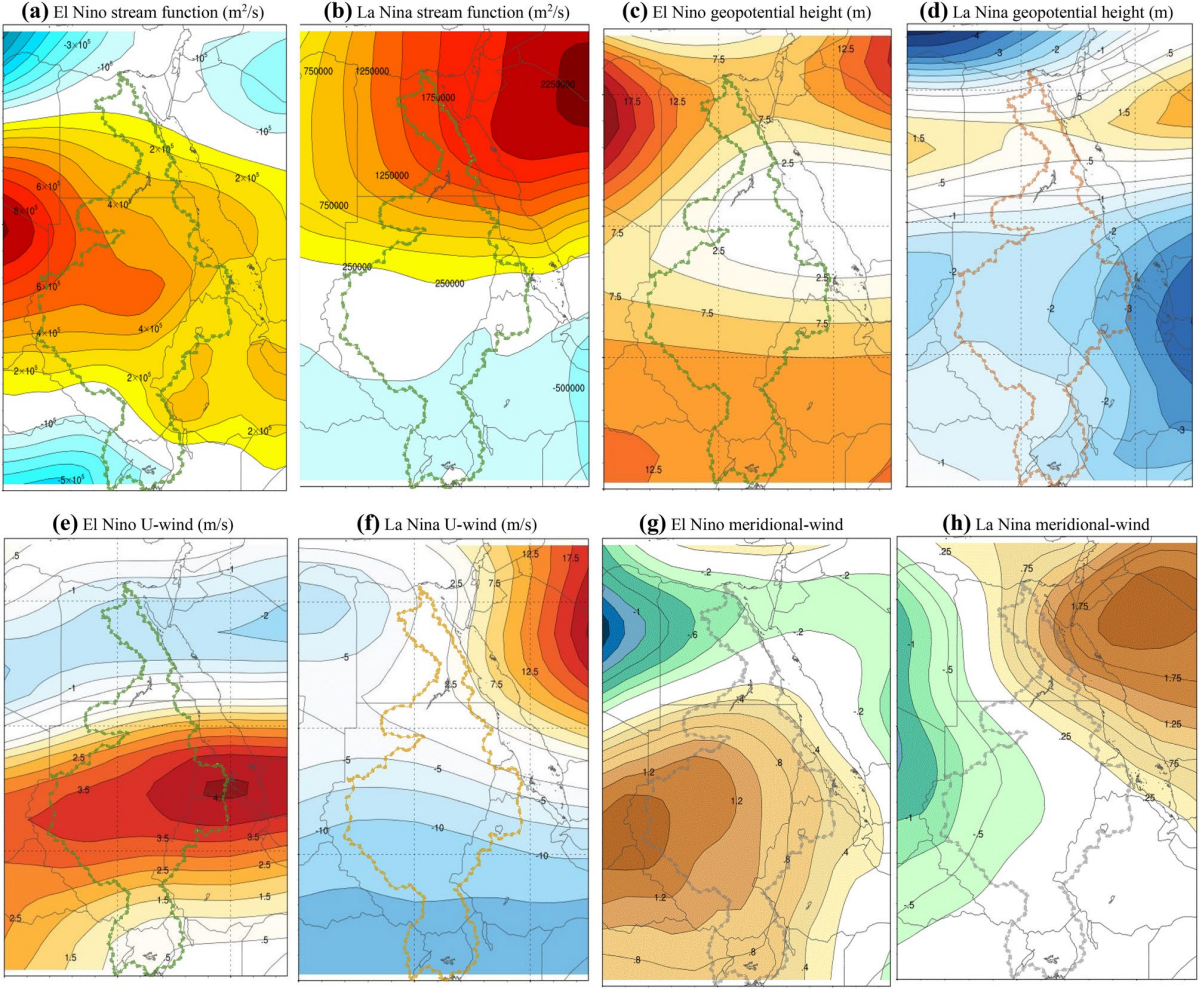
The effect of ENSO to the atmospheric circulation over NRB shown by the 750-mb stream function anomaly associated with El Nino (**a**) and La Nina (**b**) episodes (shaded contours, red/brown for positive and blue for negative anomalies), 300-mb geopotential height anomaly associated with El Nino (**c**) and La Nina (**d**) episodes, zonal and meridional wind anomaly patterns associated with El Nino (**e**,**g**) and La Nina (**f**,**h**) episodes, respectively. The signals between ENSO and GPH, stream functions, and meridional/zonal wind in the troposphere demonstrates the teleconnection between El Niño and the atmospheric circulation over of NRB. The warming over WIO and stronger El Niño, the southward shift of the lower atmospheric stream functions and meridional winds, and the westward shift of zonal winds have together contributed to worsening droughts observed in NRB in recent years. The maps were generated with NCAR Command Language (NCL) Version 6.2.1 (http://www.ncl.ucar.edu/).

To better understand changes to the atmospheric circulation over NRB, we analyzed the responses of stream function fields, GPH, and zonal/meridional winds to climate warming and ENSO (Fig. 11a–h). Associated with El Niño (La Niña) events, the 750-mb stream function show positive (negative) stream function anomalies, which correspond to high (low) GPH anomalies. The anomalous, anticyclonic stream function pattern is associated with ENSO warming, that propagates from the northwest towards southern and eastern parts of NRB during El Niño events (Fig. 11a), controlling the circulation of air mass, heat, and moisture in the NRB. During La Niña events (Fig. 11b), north-eastern anticyclone wave originates over the Arabian Peninsula and travelling west over the Red Sea and part of the Indian ocean towards Egypt, Libya, and part of Sudan, with negative stream function anomalies only over the BNB, Tanzania, and Uganda. Changes in stream function patterns are attributed to more El Niño and fewer La Niña events occurring after 1970s. Figure 11c–h shows the composite 300-mb GPH and zonal and meridional wind anomaly patterns associated with El Niño and La Niña events, respectively.

Similarly, the composite map of GPH anomalies at 300-mb during El Niño events is characterized by spatially persistent, positive anomalies, and shows an intensive anticyclonic flow, which is advective dry air, from the north (Arabian Peninsula) westward towards the NRB. In particular, the Ethiopian highlands, the Sudd region in South Sudan, Eritrea, Uganda, and the Aswan high dam of Egypt showed the highest positive GPH anomalies during El Niño events (Fig. 11c). In contrast, GPH anomalies during La Niña events show mainly negative anomalies except in the Arabian Peninsula, (Fig. 11d). Figure 11c,d implying that El Niño induced positive GPH anomalies are more consistent spatially than the negative anomalies during La Niña events. This can be attributed to the thermal inertia associated with El Niño events, i.e., El Niño induced positive GPH anomalies are more persistent and are intensified by warming. This finding indicates a warmer lower troposphere, resulting in higher surface temperature and drier weather (lower dew points) in NRB.

Differences in U-wind (meridional winds) patterns between (Fig. 11e–h) show an enhanced, positive zonal U-wind and meridional winds flow anomaly during El Niño events. Figure 11a–d also shows the shift in zonal winds (westward) and meridional winds (southward) associated with stronger El Niño events, which have contributed to the increased aridity of NRB after 1970s and resulted in a reorganization of the atmospheric circulation over NRB. Changes in regional atmospheric circulations based on stream function, GPH and U-wind anomalies associated with El Niño events further demonstrates that more persistent and stronger El Niño has resulted in drier NRB. For instance, the zonal U- wind patterns in Fig. 11e, flow from the northwest toward NRB as anomalous anticyclone waves, moving drier air continuously into the NRB and tend to be very strong over the Ethiopia’s lowland, Uganda, Burundi, the Sudd region in South Sudan, the Roseries, Eritrea, and Congo. Beside the observed change in zonal wind pattern, meridional wind anomalies associated with El Niño events shifted south (Fig. 11g). This observed changes in wind patterns associated with stronger El Niño events (Fig. 11e,g) have contributed to the abrupt changes in the NRB’s hydroclimate as shown by DCCA, WTC, and the spatial correlation between El Niño and NRB’s hydroclimate.

### Response of NRB’s hydroclimate and drought to future projection of ENSO and IOD.

**Figure 12:**
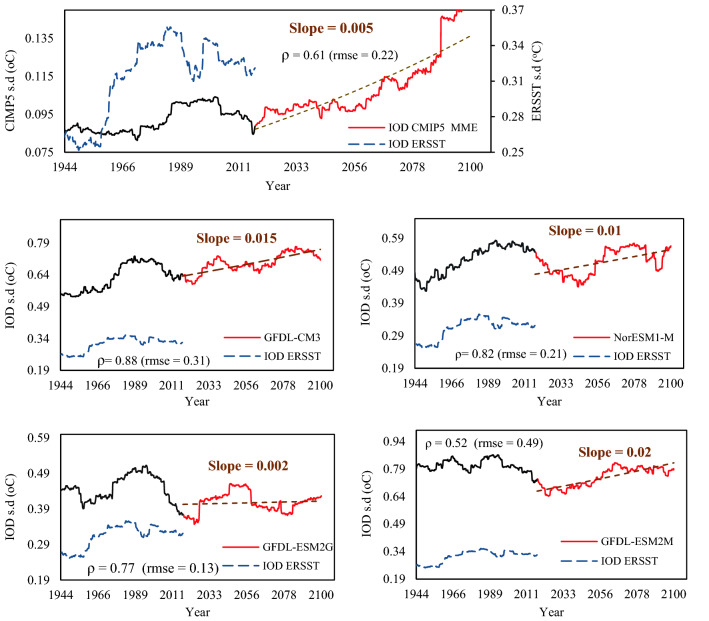
Time variation of simulated IOD amplitude. (**a**) the multi-model ensemble (MME) of the IOD. The MME IOD was computed as the difference between West (50° E–70° E and 10° S–10° N) and Eastern (90° E–110° E and 10° S–0° N) SST of the Indian ocean from 34 climate models. The 30-year running IOD amplitudes from ERSST over the period 1913–2017 are also shown (blue lines). There is significant correlation between the IOD calculated from the MME and ERSST observations (ρ = 0.61).

**Figure 13:**
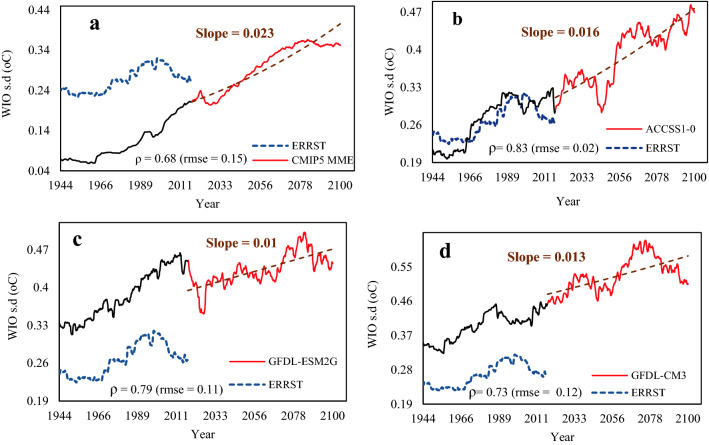
Time variation of simulated WIO amplitude. (**a**) the multi-model ensemble (MME) of the WIO amplitude from 34 climate models computed over 30-year running periods from 1913 to 2100. The WIO from the MME was calculated as area weighted average of the Indian Ocean SST over the Arabian sea (50° E–70° E and 10° S–10° N) in each model simulations. The 30-year running WIO amplitudes from ERSST over the period 1913–2017 are also shown (blue). Pearson correlation coefficients between the best models and observations (ERSST data), are displayed. The slope is the linear trend estimated at the 5% level based on the Mann–Kendall test. WIO estimated from the GCM that best agrees with the observed WIO were selected. The correlation between the MME of WIO obtained from GCMs and WIO estimated from ERSST data range between ρ = 0.61 and ρ = 0.88 (**a**–**d**). In (**a**–**d**), the variability (s.d.) of WIO has increased since the 1970s in both the observed dataset and the MME of 34 GCMs’ simulations for the historical run, which means that the western Indian Ocean has become warmer, and projected to be much warmer, at a warming trend of 0.01–0.023 °C/decade between 2019 and 2100.

The multi-model ensemble (MME) of IOD, ENSO, and WIO computed from the simulations of 34 global climate models (GCMs) of CMIP5^6^ over 1900–2100 was computed over 30-year periods. IOD, ENSO, and WIO estimated from the GCM that best agrees with the observed IOD, ENSO, and WIO were only used in this study. The selected GCMs simulate the frequency of El Niño, La Niña, IOD, WIO events for the twentieth century with some differences but are generally in reasonable agreement with observations (Figs. 12 and 13). The correlation between MME of WIO and IOD obtained from GCMs’ projections and WIO and IOD estimated from ERSST data range between ρ = 0.61 and ρ = 0.88. Figure 12a–d shows projected increasing trends of 0.01–0.02 °C/decade in IOD over 2019–2100, which could double the increasing trend in IOD observed between 1993 and 2018. As future positive IOD events are projected by GCMs to be more extreme because of global warming, NRB could suffer from more severe droughts which could occur more frequently in the future. The results also show that the variability (s.d.) of WIO has increased since the 1970s in both the observed dataset and the MME of 34 GCMs’ simulations for the historical run, which means that WIO has become warmer in recent decades, and it is projected to be considerably warmer at a trend of 0.01–0.023 °C/decade between 2019 and 2100 (Fig. 13). The warmer WIO, the more intensive El Niño, and atmospheric circulation shift in recent years are expected to play a major role modulating the future climatic conditions of NRB, likely resulting in less precipitation, RH, SMC, and the Nile flow, as warming continues in NRB over the 21st Century. Under the projected increase in WIO, future drought conditions of NRB are expected to worsen.

**Figure 14:**
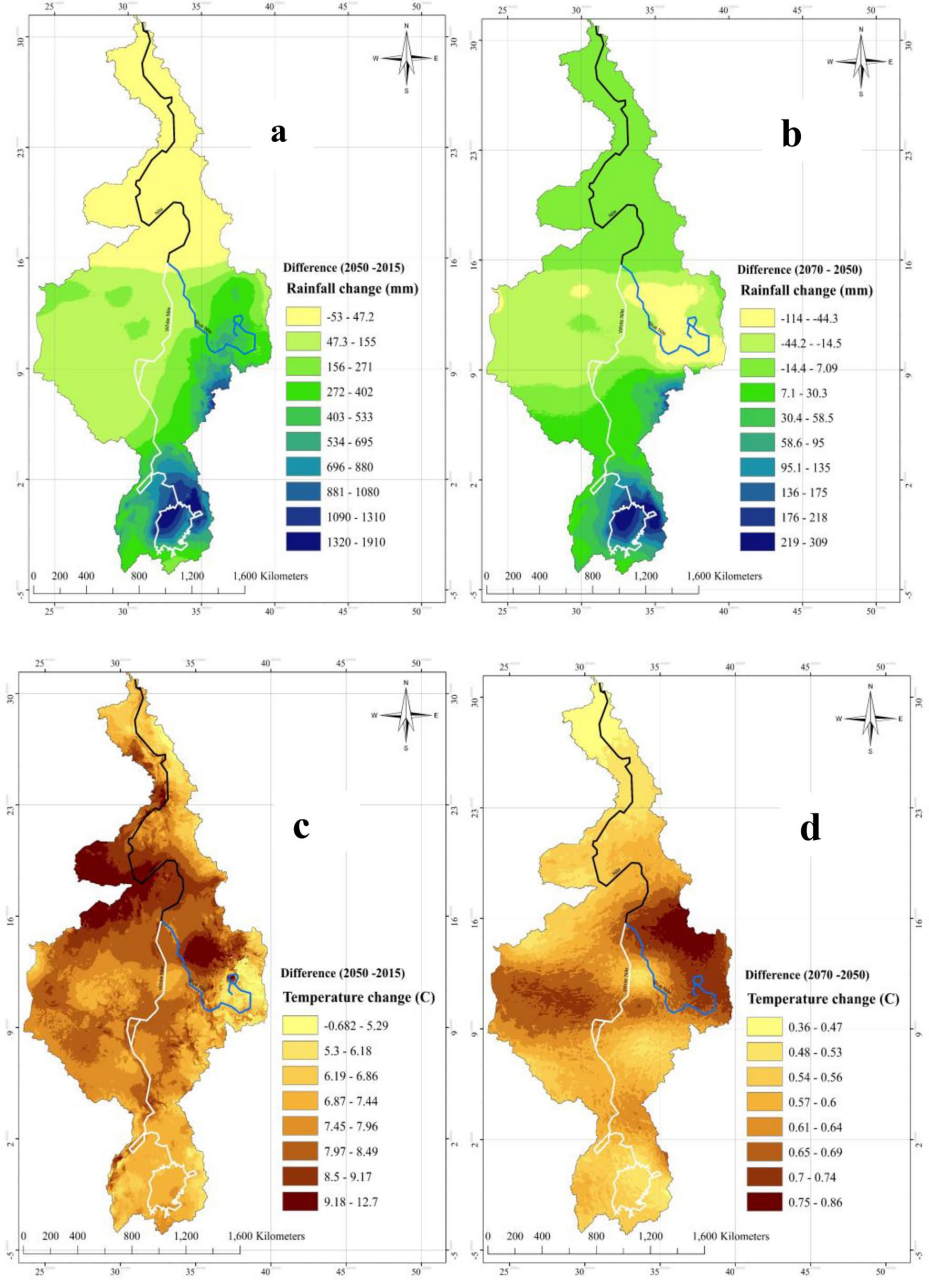
Future change of NRB’s annual precipitation (**a**) and surface temperature (**c**) in 2050, NRB’s annual precipitation and surface temperature in 2070 (**b**,**d**). In (**c**–**d**), warming trend over Sudan and Egypt will increase and the projected increase in mean temperature in this period is 6.82 °C between 2015 and 2050, while between 2050 and 2070 warming trend will continue but at a lower rate than the previous period as the difference between mean temperature in 2050 and 2070 is 0.79 °C. The increases of mean temperature in Ethiopia and Eritrea between 2015–2050 and 2050–2070, is about 6.08 °C, and 0.56–0.58 °C, respectively. While in Kenya, Tanzania, Rwanda, Burundi, Uganda, and Congo increase in mean temperature is 5.5 °C between 2015–2050 and 0.48 °C between 2050 and 2070. The maps were generated with ArcMap Version 10.1 (http://www.esri.com/en/arcgis/arcgis-for-desktop/).

**Figure 15:**
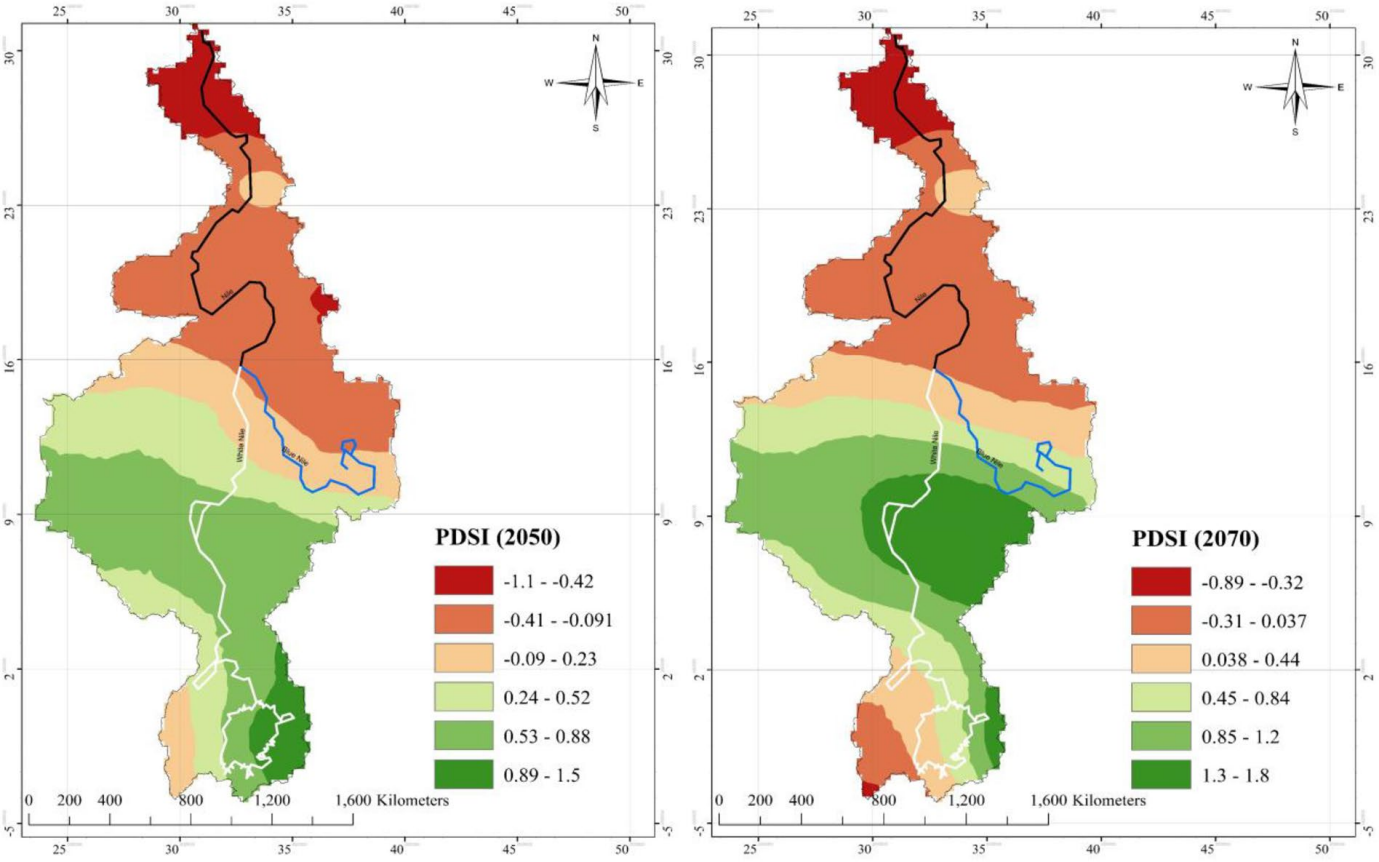
Future projection of drought severity ‘PDSI’ 2050,2070. According to sc-PDSI projection, large parts of Egypt and Sudan could suffer mild to moderate hydrologic droughts over the 21st Century, and countries such as Kenya, Tanzania, Rwanda, Burundi, Uganda, and Congo are projected to suffer incipient droughts. Major areas of Egypt and Sudan is projected to have mild to moderate drought, this can be explained by the very high increase in projected Ts. Between 2050 and 2070 as the increase in mean temperature is lower than the previous period and precipitation increase is also lower, the entire region is covered by incipient drought (mainly Egypt and Sudan-except the White Nile region) and slightly wet to moderately wet in Kenya, Tanzania, Rwanda, Burundi, Uganda, and Congo and Ethiopia. This reflects the effect of warming rate in future drought condition. The maps were generated with ArcMap Version 10.1 (http://www.esri.com/en/arcgis/arcgis-for-desktop/).

To estimate the influence of the projected increase in IOD, WIO, and El Niño on the NRB’s hydroclimate and droughts, future projections of NRB’s hydroclimate and droughts were also analyzed (Figs. 14 and 15 and supplementary Fig. 16). Similar to the approach adopted in computing the MME of IOD, ENSO, and WIO, we have selected the most suitable GCMs that simulate the NRB’s hydroclimate. The selection process was limited to the GCMs that best agrees with the observed IOD, ENSO, and WIO. We have also used the GCMs that were identified in previous study by Siam and Eltahir^37^. These GCMs were bias corrected to minimize the impact of discrepancies between simulations and observations. Based on the analysis of simulations of 34 GCMs of CMIP5 , the warming of NRB is projected to be at 0.24 °C/decade (0.36 °C/decade) (Fig. 14 and supplementary 16a), annual precipitation is projected to decrease at about 16.5 mm/decade, RH is projected to decline at 0.87% /decade (1.04% /decade), PET is projected to increase at 11.4 mm/decade (18.4 mm/decade), and monthly SMC is projected to decrease at 0.12 mm/decade (0.72 mm/decade), under Representative Concentration Pathways RCP 2.6 (RCP4.5) scenarios over 2020–2050, respectively. With high spatial variability in precipitation, the annual precipitation in Egypt and Sudan could decline by 15.5 mm/decade but for the White Nile region, it could increase by 28.9 mm/decade over 2020–2050 (supplementary Fig. 16b). Between 2050 and 2100, the warming trend will continue but projected at a lower rate, annual precipitation is projected to increase at 5 mm/decade, RH is projected to only decline at 0.23%/decade (0.34%/decade), monthly SMC is projected to decrease at 0.65 mm/decade (2.5 mm/decade), and PET to increase by 10 mm/decade (14.8 mm/decade) under RCP 2.6 (RCP4.5), respectively. It seems that agricultural drought of NRB will get worse over the 21st Century. Climate models project more frequent and stronger El Niño events between 2020 and 2100^16,38^, leading to worsening droughts and more severe surface drying in NRB over the twenty-first century. Furthermore, large parts of Egypt and Sudan are projected to suffer mild to moderate hydrologic droughts over the 21st Century (Fig. 15), and countries such as Kenya, Tanzania, Rwanda, Burundi, Uganda, and Congo are projected to suffer incipient droughts.

## Conclusions and implications

The results of this study have brought new insight on the hydroclimate variability of NRB, provide a clearer perspective on the driving mechanisms behind the hydroclimate variations and worsening droughts of NRB in recent decades, and have corrected some results reported in past studies. Low precipitation and climate warming have been attributed as key driving forces to droughts of Africa^1–5^. Our analysis of hydroclimate data demonstrated climatic changes that contributed to recent increasing aridity of NRB: a decreasing trend in annual precipitation at 16.2 mm/decade since 1970s, increasing trend in wind speed and zonal wind stress at 0.02 m/decade and 1.51 m^2^/s^2^/decade respectively since 1975, increasing trend in geopotential height (GPH) at 3.1 m/decade since 1976, warming trend at 0.19 °C/decade, decreasing trend in relative humidity (RH) since 1977, decreasing trend in soil moisture content (SMC) and groundwater storage (GWS) at 0.84 mm/decade and 1.44 mm/decade, respectively since 1979. This is evident in the decreasing trend in precipitation, RH, SMC, and TWS, and increasing trend in wind speed, wind stresses, GPH, Ts, and AET. The increase in AET is also shown by other studies, that higher AET is related to higher wind speed and wind stresses, warming and lower RH^39,40^. These observed changes are strongly linked to El Niño and IOD. Our results demonstrate that warming, El Niño and IOD have played a crucial role on NRB’s inter-decadal hydroclimate variability, but IOD has played a more important role in modulating NRB’s hydroclimate at higher timescales than El Niño.

Beside the observed warming, El Niño -driven changes to wind patterns have also contributed to a drier NRB in recent years. A westward shift in zonal winds and southward shift in meridional winds, caused an enhancement in the blocking pattern, with strong anticyclonic waves of dry air that keeps moving dry air over the Roseires, lowlands of Ethiopia, the Sudd region in South Sudan, Eritrea, Congo, Uganda, and Burundi. This pattern controls the circulation of air mass, heat, and moisture fluxes in NRB, which explain the observed changes in NRB’s hydroclimate. In other words, changes in atmospheric circulations over NRB due to the southward shift of the lower atmospheric stream functions, GPH, El Niño-wind patterns has resulted in the observed changes in NRB’s hydroclimate and intensified drought. This is also evident in the WTC and DCCA between AET and wind stresses, which shows that wind stresses have had a positive (negative) influence on NRB’s AET (RH), i.e. The increase in wind stresses lead to increase (decrease) in AET and surface temperature (RH). Andresen et al.^41^ showed a similar effect of El Niño -driven changes to wind patterns in the United States, lower precipitation, and significant warming. Lastly, warming of WIO and stronger El Niño and IOD, have together contributed to worsening droughts observed in NRB in recent years, where its flow at upstream and downstream stations have decreased by 13.7 m^3^/s/decade (upstream) and by 114.1 m^3^/s/decade after 1964. Climate projections suggest that under the combined impact of warming and stronger WIO and El Niño episodes, future droughts of the NRB will worsen.

## Methods

### Observational datasets

Historical monthly temperature, relative humidity, and specific humidity data for 1900–2017 were taken from the 20th Century Reanalysis V2 Dataset, the 20th Century Reanalysis V2 data provided by NOAA/OAR/ESRL (https://www.esrl.noaa.gov/psd/). Temperature anomaly data for 1910–2017 was taken from the HadCRUT4 global temperature dataset developed by the Climatic Research Unit of University of East Anglia (CRU.Ts4.03)^42^ in conjunction with the Hadley Centre (UK Met Office). Historical observed monthly precipitation data for 1948–2017 was taken from the University of Delaware precipitation dataset (Udel.^43^) developed from a large number of climate stations of the Global Historical Climate Network, We have also analyzed precipitation data from the following datasets (1) the 20th Century Reanalysis V3 Dataset provided by the NOAA/OAR/ESRL PSL, Boulder, Colorado, USA (20CRv3), (2) the University of East Anglia’s Climate Research Unit (CRU.TS4.03) https://crudata.uea.ac.uk/cru/data/hrg/cru_ts_4.04/, (3) the 0.25° Gridded data of Global Precipitation Climatology Center (GPCC v2.2), (iv) GPCC Full Data Monthly Product Version 2018 extended with GPCC Monitoring Monthly Product version 6 (GPCC v.6) (https://opendata.dwd.de/climate_environment/GPCC/html/download_gate.html), (v) JRA-55 reanalysis dataset, and (vi) NCEP/NCAR Reanalysis dataset. Monthly Geopotential height, zonal and meridional wind stresses, and wind speed from 1948 to 2017 were drawn from the National Centers for Environmental Prediction– National Center for Atmospheric Research (NCEP–NCAR) reanalysis 1 (NCEP-R1) and the 40-yearr European Center for Medium-Range Weather Forecasts (ECMWF) Re-Analysis (ERA-40). Digital elevation model (DEM) of 30 m resolution was obtained from the Global Elevation Model (GDEM) version 2 databases (http://aster.usgs.gov). The Nile River hydroclimate from these datasets were calculated as the average of all grid boxes within the basin boundary from each dataset.

Monthly precipitation and temperature data in gridded form (0.5° × 0.5°) from 1948 to 2017 was obtained from the Global monthly precipitation and temperature data of the Princeton global forcings^44^. These datasets are of the observational-reanalysis hybrid type developed from a combination of datasets, which include the NCEP–NCAR reanalysis dataset^45^, the TRMM dataset, the CRU TS2.0, the GPCP, and the NASA Langley Research Center SRB products^44^. These are credible datasets are widely used in climatology studies due to their robustness for variability analyses^46–48^. Total atmospheric water vapor content was extracted from the MODIS atmosphere profiles product (MOD07), and emissivity data were derived from averaging MODIS-bands 31 and 32, while the land cover map taken from the global land cover of Africa archive of 2008 and was updated using available Landsat images (http://www.africover.org/index.htm). Land cover map was derived from the global land cover of Africa archive of the year 2008 and was updated using available Landsat images. (http://www.africover.org/index.htm), long term observed monthly Nile River flow data were collected from three stations, the monthly flows at Dongola station-Sudan, Aswan dam station (1900–1984), and the Blue Nile station in Khartoum from 1900 to 1984 were extracted from the Global River Discharge Database (RivDIS v1.1), in addition we obtained the monthly flows at the Blue Nile station from recorded measurements between 1984 and 2012.

Area-averaged of TWS were computed from water balance model based on GLADS-CLSM025 TWS between 1948and 2017 and the Gravity Recovery and Climate Experiment (GRACE) between 2003 and 2017. NRB’s soil moisture data were computed as an area average of ERA-Interim, CLM v.4, FLDAS, WaterGAP model, and GLDAS soil moisture datasets. Long-term runoff observation for the Nile River basin was extracted from the GRUN- Runoff observation-based global gridded runoff dataset^49^ from 1902 to 2014. This dataset was newly developed for climatological, hydrological, and environmental studies and are close to near natural runoff conditions and represent the excess of water available to ecosystems. Warm spell duration, the annual number of days contributing to events where 6 or more consecutive days experience a daily maximum temperature TX > 90th percentile was extracted from the HadEX2 observational data set. CMIP5 RCP2.6, RCP4.5, and RCP8.5 experiments over the period 1900–2100 from 34 global climate models (GCMs) of CMIP5. The models included ACCESS1-0, ACCESS1-3, CCSM4, CNRM-CM5, CSIRO-MK3-6-0, FGOALS-g2, GFDL-CM3, GFDL-ESM2G, GFDL-ESM2M, GISS-E2-R, HadGEM2-CC, HadGEM2-ES, IPSL-CM5A-LR, IPSL-CM5A-MR, IPSL-CM5B-LR, MIROC5, MIROC-ESM, MPI-ESM-LR, MPI-ESM-MR, MRI-CGCM3, NorESM1-M and NorESM1-ME.

### Statistical analysis, trends, and change point of NRB’s hydroclimate

We first did a detailed analysis of monthly precipitation, surface temperature, geopotential height, relative humidity, specific humidity, potential and actual evapotranspiration, wind speed, zonal and meridional wind stresses data of the NRB, Nile flow, surface runoff, SMC, and TWS data. These data were first checked to ensure quality control and homogenization, downscaled to the NRB, then the non-parametric statistic of Pettitt’s test^50^ and the modified-Mann–Kendall^51^ codes were written in R-software and employed to detect abrupt changes and trends in these variables. Pettitt’s test is defined as:

1 $$K_{T} = max\left| {U_{t} ,T} \right|$$

where,

2 $$U_{t} ,T = \mathop \sum \limits_{i = 1}^{n - 1} \mathop \sum \limits_{j = t + 1}^{n} sgn\left( {x_{j} - x_{i} } \right)$$

$K_{T}$ is the detected change-point of the series if it is statistically significant. The *p*-value of $K_{T}$ is approximated by:

3 $$p \approx 2e^{{{\raise0.7ex\hbox{${ - 6K_{T}^{2} }$} \!\mathord{\left/ {\vphantom {{ - 6K_{T}^{2} } {T^{3} + T^{2} }}}\right.\kern-\nulldelimiterspace} \!\lower0.7ex\hbox{${T^{3} + T^{2} }$}}}}$$

The non-parametric Mann–Kendall test statistic is calculated according to:

4 $$S = \mathop \sum \limits_{i = 1}^{n - 1} \mathop \sum \limits_{j = i + 1}^{n} sgn\left( {x_{j} - x_{i} } \right)$$

Trend is estimated for a time series $x_{i} ,$ i = 1, 2…n−1 and $x_{j }$, j = i + 1, i + 2…n. Each $x_{i}$ is a reference and compared with remaining data points $x_{j}$(see 5):

5 $$Sgn\left( {x_{j} - x_{i} } \right) = \left\{ {\begin{array}{*{20}c} { + 1\,if\; \left( {x_{j} - x_{i} } \right) > 0 } \\ {0\,if\;\left( {x_{j} - x_{i} } \right) = 0} \\ { - 1\,if\; \left( {x_{j} - x_{i} } \right) < 0} \\ \end{array} } \right.$$

The variance statistic is estimated as:

6 $$Var \left( S \right) = \frac{{n\left( {n - 1} \right)\left( {2n + 5} \right) - \mathop \sum \nolimits_{j = 1}^{p} t_{j} \left( {t_{j} - 1} \right)\left( {2t_{j} + 5} \right)}}{18}$$

where p is the number of groups in which each group consists of data points of equal values, and t_j_ is the number of data points in the jth tied group. The statistic S is approximately normal distributed by the following Z-transformation:

7 $$Z = \left\{ {\begin{array}{*{20}c} {\frac{S - 1}{{\left( {Var \left( S \right)} \right)^{0.5} }}\,if\; S > 0 } \\ {0\,if \;S = 0} \\ {\frac{S + 1}{{\left( {Var \left( S \right)} \right)^{0.5} }}\,if \;S < 0} \\ \end{array} } \right.$$

The slope (Tj) is computed according to Sen^52^ as follow:

8 $$T_{j} = \frac{{x_{j} - x_{i} }}{j - 1}$$

To ensure accurate detection of change point in the NRB’s hydroclimate, we have written R-codes for seven other change points detection methods (Bayesian change point detection (BCP), non-parametric multiple change-point analysis (MCP), lepage sequential and batch change detection method (CPM), optimal multiple change point algorithms (PELT), structure change features method (SCFM), bayesian information criterion (BIC), and segmentation by Dynamic Programming (DP)) and applied them to the NRB’s hydroclimate. Unlike Pettitt's test, BCP method given in Wang and Emerson^53^ provides a tool for evaluating the strength of abrupt changes “posterior probability” at each point of the time series, where points with the highest posterior probability are considered true change points. In another word, the detection rate of BCP depends more on the magnitude of change than other methods. Similarly, BIC method provides a strongly consistent selection of the optimal number of change points in a timeseries^54^. BIC is derived from an asymptotic expression of the Bayes factor, therefore, has been applied straightforwardly in change-point models. In the MCP method given in Matteson and James^55^, the estimation of the most likely locations of change point within the timeseries is based on a hierarchical clustering using the energy statistics. CPM is an approach to sequential change detection, which allows standard statistical hypothesis tests to be deployed sequentially to detect single and multiple change points^56^. PELT given in Killick et al.^57^ estimates multiple change points using penalization. The main drawback of this method is that it requires a user specified penalty term. In addition to the above methods, we have also applied the SCFM method to detect the change point in the NRB’s hydroclimate. The main difference between above methods and SCFM is that SCFM provides confidence intervals of change points like Pettitt's test. Lastly, the DP method described in Muggeo^58^ was used to further confirm the locations of the estimated change points.

### Surface energy balance algorithm

The FAO-56 Penman–Monteith method^59^ was used to model reference evapotranspiration on a grid-by-grid basis. Then, surface energy balance data were used to estimate actual evapotranspiration of NRB for 1912–2018, and from which their variability and anomalies were analyzed. To estimate AET, first the net solar radiation (Rn), NDVI, albedo, roughness length, and soil heat flux (G) were calculated in ArcGIS 10.1. ESRI’s ArcGIS. Then the surface energy balance algorithm was employed to model AET based on the approach given in Bastiaanssen et al^60^.

### Drought detection methods

The frequency, intensity, change point and trend of the meteorological, agricultural, and hydrological droughts of NRB under the effect of climate change were investigated based on the SPI, NDVI, SPEI, sc-PDSI, and runoff anomaly index, estimated, respectively. SPI computation was made based on the method proposed by McKee et al.^61^ and Edwards and McKee^62^. This computation was made to drive SPI at different scale at 1, 3, 6, 12 and 48-month. The SPEI at 1, 3, 6, 12 and 48-month timescales over 1940–2018 was computed for the NRB from five precipitation datasets (Udel., GPCC, 20CRv3, CRU.TS4.03, and GPCC V2018) as the monthly difference between precipitation (Pr) and PET. For example, for the month i, $SPEI_{i} = Pr_{i} - PET_{i}$, where $Pr_{i}$ is the monthly precipitation obtained from GPCC, 20CRv3, CRU.TS4.03, JR-55, NCEP/NCAR-R1, and GPCC V2018 and $PET_{i}$ is the monthly PET based on FAO-56 Penman–Monteith method^59^. This index have a crucial advantage over other drought indices that consider the effect of PET on drought severity due to its ability to identify different drought types and the impacts of global warming. NDVI data for the year 2002–2017 was extracted from the U.S. Geological Survey (USGS) Earth Resources Observation and Science (EROS) Center "eMODIS" products. The theory behind the popularity of NDVI in agricultural drought studies comes from its dependency on the near infrared reflectance (NIR) from vegetation cover and the visible-red reflectance (RED). Where NDVI = (NIR−RED)/(NIR + RED), these two reflectance components measure the density of chlorophyll contained in vegetative cover. NDVI change detection model was developed in ArcGIS model builder to capture the change in NDVI through time and identify areas with agricultural droughts. The sc-PDSI was obtained from Climate Analysis Section of the National Center for Atmospheric Research in a gridded format and was used as a reference for actual sc-PDSI calculation. The computation of the sc-PDSI was made using the sc-PDSI package in R-software and is based on the approach adapted in Wells et al.^63^. Runoff anomaly index was calculated from the long-term runoff observation for the Nile River basin between 1902 and 2014.

### Wavelet analysis and wavelet coherence

We have written R-code for the Morlet wavelet analysis and used it to investigate the temporal variability, periodicities, and the cyclic behavior of NRB’s hydroclimate. To evaluate the possible impacts of El Niño and IOD on the hydroclimate of the NRB, wavelet coherence was also used to estimate the spatio-temporal correlation field between these hydroclimate variables and El Niño and IOD. The wavelet power spectra of each time series were calculated as follow:

9 $$W_{{x,{\Psi }}} \left( {s,t} \right) = \left( {X \left( t \right)*{\Psi }_{s} \left( t \right)} \right)$$

where *t* is the time series, ${\Psi }_{s}$ is the Morlet mother wavelet at the scale *s*. The Morlet mother wavelet can be defined as follow:

10 $${\Psi }_{s} \left( \eta \right) = {\uppi }^{{{\raise0.7ex\hbox{${ - 1}$} \!\mathord{\left/ {\vphantom {{ - 1} 4}}\right.\kern-\nulldelimiterspace} \!\lower0.7ex\hbox{$4$}}}} e^{{\iota \omega_{s} \eta }} e^{{{\raise0.7ex\hbox{${ - \eta^{2} }$} \!\mathord{\left/ {\vphantom {{ - \eta^{2} } 2}}\right.\kern-\nulldelimiterspace} \!\lower0.7ex\hbox{$2$}}}}$$

where, $\eta$ is time, $\omega_{s}$ is frequency, and $\iota$ (imaginary number) which is the square root of minus one.

We have also analyzed the phase difference between NRB’s hydroclimate (drought indices and flow), El Niño and IOD using the methodology of Torrence and Compo^64^. The phase difference provides information about the possible delay in the relationship between NRB’s hydroclimate (drought indices and flow) and El Niño and IOD. The WTC coefficient is given as:

11 $$R^{2} \left( {s,t} \right) = \frac{{\left| {S\left( {s^{ - 1} W_{xy} \left( {s,t} \right)} \right)} \right|^{2} }}{{S\left( {s^{ - 1} \left| {W_{x} \left( {s,t} \right)} \right|^{2} } \right)*S\left( {s^{ - 1} \left| {W_{y} \left( {s,t} \right)} \right|^{2} } \right)}}$$

where x is the hydroclimate variable (drought indices or flow) been analyzed and y is El Niño or IOD. t is the dimensionless time-shift parameter, $W_{xy} \left( {s,t} \right)$ is the cross wavelet transform of the two-time series, $W_{x }$ and $W_{y}$ are the sums of ranks of observations in *x* and *y*, respectively, and S is a smoothing operator, which was calculated based on the approach of Torrence and Compo^64^.

### Composite analysis and spatial correlation

To investigate the hydroclimate variability and the spatio-temporal changes in the NRB’s hydroclimate, composite maps of precipitation, Ts, GPH, RH, specific humidity, scalar wind, meridional and zonal wind, soil moisture, surface runoff, and PET data were derived by the difference between data of 1985–2017 and data of 1948–1984. These composite maps were computed as the ratio of the mean seasonal precipitation, Ts, GPH, RH, specific humidity, scalar wind, meridional and zonal wind, soil moisture, surface runoff, and PET for March–May (MAM), June–August (JJA), September–November (SON) and December–February (DJF) seasons of 1948–2017 in anomalous (El Niño) years relative to the corresponding long term mean fields, respectively. To demonstrate the effects of El Niño on the NRB’s climate, we have only considered years with strong El Niño activity (1958, 1982, 1983, 1987, 1992 and 1997, 2005) in the composite analysis. Composite analysis of the NRB’s hydroclimate data between 1948 and 2017 and sc-PDSI, monthly temperature, temperature anomaly, monthly precipitation and precipitation anomaly were also analyzed for each riparian country of NRB, to relate climate warming to trend and change points in hydrologic droughts of these countries.

To analyze the relative influence of IOD and ENSO, we have further estimated the spatial correlation between NRB’s hydroclimate and El Niño and IOD to better explain in greater details the teleconnection of ENSO and IOD to each riparian country of NRB using the independent composite method adopted in Saji and Yamagata^28^. This method was used because we have found a strong coupling between IOD and ENSO, and there is a need to study in detail the separate influence of IOD and ENSO in the NRB’s hydroclimate. First, we have removed the co-occurring IOD, and ENSO events from the composite to remove IOD(ENSO) influence on the NRB’s hydroclimate, then years with independent IOD (ENSO) influence were used to compute independent composite maps of the NRB’s hydroclimate. Once the coupling between IOD and ENSO events was removed, we used the spatial correlation locate areas with pure IOD or ENSO influence.

### Detrended cross correlation analysis

For non-stationary data, detrended cross correlation analysis (DCCA) ensures that the results obtained are not affected by trend. Therefore, DCCA was used to investigate the role of ENSO and IOD on the NRB’s hydroclimate, flow variability, and droughts over inter-decadal and longer timescales. For instance, DCCA between IOD and El Niño amplitudes and NRB’s precipitation, Ts, GPH, RH, specific humidity, PET, AET, wind speed, zonal and meridional wind stresses, drought indices, Nile flow, surface runoff, SMC, and TWS were used to estimate the teleconnection of ENSO and IOD on NRB’s hydroclimate data divided over 20-year running periods from 1920 to 2017. The DCCA coefficients of hydroclimate variable *x(i)* and El Niño or IOD *y(i)* were calculated as follow:

12 $$X_{k} = \mathop \sum \limits_{i = 1}^{k} x_{i}\,and\, Y_{k} = \mathop \sum \limits_{i = 1}^{k} y_{i} ,\;{\text{where}}\,k = { 1}, \ldots ,N.$$

First, the hydroclimate variables, El Niño, and IOD were divided into equal length segments $(N_{n} )$. Then, using regression models, we have defined the local trend in each segment $\check{X}_{n, s} \left( k \right)$ and $\check{Y}_{n,s} \left( k \right)$, where $s = 1, \ldots ,N_{n}$. The time series data $X_{k} and Y_{k}$ are detrended by subtracting the local trends $\check{X}_{n, s} \left( k \right)$ and $\check{Y}_{n,s} \left( k \right)$ from original time-series in each segment. Next, we calculated the detrended covariance and variance function in each segment as follow:

13 $$F_{DCCA}^{2} \left( n \right) = \frac{1}{{nN_{n} }}\mathop \sum \limits_{s = 1}^{{N_{n} }} \mathop \sum \limits_{{k = n\left( {s - 1} \right) + 1}}^{ns} \left[ {X\left( k \right) - \check{X}_{n, s} (k} \right]\left[ {Y\left( k \right) - \check{Y}_{n, s} (k} \right]$$

Then, the detrended variance of the two-time series, $f_{DF, }$ is calculated as follow:

14 $$f_{DFA, X\left( n \right) = } \sqrt {\frac{1}{N - n}\mathop \sum \limits_{i = 1}^{N - n} \left[ {{\raise0.7ex\hbox{$1$} \!\mathord{\left/ {\vphantom {1 {\left( {n - 1} \right)}}}\right.\kern-\nulldelimiterspace} \!\lower0.7ex\hbox{${\left( {n - 1} \right)}$}}\mathop \sum \limits_{k}^{i + n} \left( {X_{k} - \tilde{X}_{k,i} } \right)} \right]}$$

15 $$f_{DFA,Y\left( n \right)} = \sqrt {\frac{1}{N - n}\mathop \sum \limits_{i = 1}^{N - n} \left[ {{\raise0.7ex\hbox{$1$} \!\mathord{\left/ {\vphantom {1 {\left( {n - 1} \right)}}}\right.\kern-\nulldelimiterspace} \!\lower0.7ex\hbox{${\left( {n - 1} \right)}$}}\mathop \sum \limits_{k}^{i + n} \left( {Y_{k} - \tilde{Y}_{k,i} } \right)} \right]}$$

This calculation is repeated for all segment, if the series are power-law cross-correlated, then $V_{DCCA} \sim n^{2\tau }$. The $\tau$ exponent is the long-range power-law cross correlation between two-time series, and is calculated through linear regression of $\log \left[ {V_{DCCA} \left( n \right)} \right]$ and log n. Finally, the DCCA cross correlation coefficient $\left( \rho \right)$ was calculated according to:

16 $$\rho = \frac{{F_{DCCA\left( n \right)}^{2} }}{{f_{DFA, X\left( n \right) } f_{DFA,Y\left( n \right) } }}$$

The value of $\rho$ ranges from − 1 to 1, a value of $\rho = 0$ means there is no cross-correlation between the two-time series being analyzed. We have also studied the relationship between NRB’s hydroclimate, flow, droughts and between ENSO and IOD based on 20-year overlapping to assess stability, this method offers an opportunity to test the stationarity of the relationships over time^65^. Supplementary Table 6 and 7 show the cross-correlation coefficients with error for each hydroclimate variable and El Niño, IOD, SEIO, and WIO.

### Detection and attribution methods

The observed ENSO and Indian Ocean dipole (IOD) amplitude, and zonal and meridional winds stresses over the NRB were derived and analyzed. ENSO and IOD amplitude were estimated as the standard deviation (SD) of the Niño3.4 and IOD index over 20-, 30-, 40- and 50-year windows from 1950 to 2017 using the ERSST data sets. Zonal and meridional wind stresses amplitude (10^−1^ N m^−2^) were calculated as the SD of zonal and meridional winds stresses over 20-, 30-, 40- and 50-year windows from 1950 to 2017, using NCEP/NCAR data sets of 1950–2017. Then, composite analysis, DCCA and WTC were used to investigate the role of ENSO and IOD on the NRB’s hydroclimate variability and drought severity over inter-decadal and longer timescales. Furthermore, to investigate changes in regional atmospheric circulation we analyzed the responses of stream function fields, GPH, and zonal / meridional winds to climate warming and ENSO. For the March–April–May (MAM), June–July–August (JJA), September–October– November (SON) and December–January–February (DJF) seasons of 1950–2017, stream function fields, GPH, and zonal/meridional winds composites were computed as the ratio of mean seasonal stream function fields, GPH, and zonal/meridional winds in anomalous (El Niño) years relative to the long term mean seasonal stream function fields, GPH, and zonal/meridional winds. To emphasize the effect of El Niño on seasonal stream function fields, GPH, and zonal/meridional winds, years with strong El Niño activity (1958, 1982, 1983, 1987, 1992 and 1997, 2005) were only considered in the composite analysis.

To better understand hydrologic droughts of NRB, we also investigated the Nile River flow variability and the teleconnection of ENSO and the dipole mode to Nile flow over 1912–2012. The NRB flow are computed over 30-year running periods from 1913 to 2010 for the Blue Nile station, and from 1913 to 1984 for Dongala station. El Niño 3.4, IOD, SEIO, WIO amplitudes are computed as the SD of the El Niño 3.4, IOD, SEIO, WIO indexes over 30-year windows from 1913 to 2017, using the ERSST data sets. We have also analyzed the projected WIO and IOD using climate projections of 34 global climate models (GCMs) of CMIP5. Future projection of WIO and IOD were analyzed based on climate projections of 34 global climate models (GCMs) of CMIP5. First, WIO was estimated as areally weighted SST simulated by each GCM over the region (50° E–70° E and 10° S–10° N) as described in Saji et al.^36^. Then, the multi-model ensemble (MME) of the WIO computed from the simulations of 34 GCMs was computed over 30-year periods from 1913 to 2100. WIO estimated from the GCM that best agrees with the observed WIO was selected. Furthermore, projections of IOD between 2019 and 2100 were computed as the difference between Western (50° E–70° E and 10 °S–10° N) and Eastern (90° E–110° E and 10° S–0° N) SST of the Indian ocean simulated by the 34 GCMS. Lastly, based on RCP scenarios of 34 GCMs of CMIP5^6^, future changes to the annual precipitation, temperature, PET, SMC, relative humidity, sc-PDSI, El Niño 3.4 index, and IOD of NRB until 2100, and their impact to hydrological droughts of NRB were projected.

## Supplementary Information

Supplementary Information.

## Data Availability

The observational data, flow data and remote sensing data that support the findings of this study are available from the corresponding author upon reasonable request.

## Abbreviations

AET: Actual evapotranspiration
BCP: Bayesian change point detection
BIC: Bayesian information criterion
BNB: Blue Nile basin
DCCA: Detrended cross correlation analysis
ENSO: El Niño southern oscillation
GPH: Geopotential height
GPCC: Global precipitation climatology project
IOD: Indian Ocean dipole
IPCC: Intergovernmental panel on climate change IPCC
CPM: Lepage sequential and batch change detection method
MK: Mann–Kendall
NCEP–NCAR: National centers for environmental prediction–reanalysis dataset
NRB: Nile River basin
MCP: Non-parametric multiple change-point analysis
NDVI: Normalized difference vegetation index
PELT: Optimal multiple change point algorithms
PET: Potential evapotranspiration
RH: Relative humidity
DP: Segmentation by dynamic programming
sc-PDSI: Self-calibrating palmer drought severity index
SMC: Soil moisture content
SEIO: Southeast Indian ocean
SPI: Standard precipitation index
SPEI: Standardized precipitation-evapotranspiration index
SCFM: Structure change features method
20CRv3: The 20th century reanalysis V3 dataset
AR5: The fifth assessment report
TWS: Total water storage
Udel.: University of delaware precipitation dataset
CRU.TS4.03: University of East Anglia’s Climate Research Unit
WTC: Wavelet coherence
WIO: Western pole of the Indian Ocean dipole

## Code availability

R-codes for abrupt changes, wavelet analysis, and attributions can be obtained from the first author upon request.
