# Supplementary material for: Worsening drought of Nile basin under shift in atmospheric circulation, stronger ENSO and Indian Ocean dipole
Source: Sci Rep. 2022 May 16;12:8049. doi: 10.1038/s41598-022-12008-8 (PMC9110430; doi:10.1038/s41598-022-12008-8)
Supplement: Supplementary file 1 — Supplementary Information. [file 41598_2022_12008_MOESM1_ESM.docx]

**Worsening Drought of Nile Basin due to Circulation Shift, Stronger ENSO, and Indian Ocean Dipole**

Shereif H. Mahmoud^1^, Thian Yew Gan^1*^, Richard P. Allan^2^, Jianfeng Li^3^, and Chris Funk^4^

^1^Department of Civil and Environmental Engineering, University of Alberta, Edmonton, T6G 1H9, Canada

^2^Department of Meteorology, University of Reading, Berkshire, RG6 6BB, United Kingdom

^3^Department of Geography, Hong Kong Baptist University, Hong Kong

^4^Climate Hazards Center, University of California Santa Barbara, CA 93106, United States of America

**Supplementary Fig.** 1: Warm spell duration (WSD) (a) and AET (b), WSB is the annual number of days contributing to events where 6 or more consecutive days experience a daily maximum temperature TX > 90^th^ percentile, averaged over the NRB using the HadEX2 observational data set. The slope is the linear trend estimated and the p-value is the trend significance based on the Mann–Kendall test.

**Supplementary Table 1:** Change point and trend analysis of climate variables in the NRB

|  |  | **Pettitt test** | | | | **Mann–Kendall test** | | | |  |  |
| --- | --- | --- | --- | --- | --- | --- | --- | --- | --- | --- | --- |
| **Nile river basin** | Years | K | t | P | trend | Tau | Sen's slope | P | Trend | Mb | Ma |
| Monthly Tm | 1900-2017 | 44607 | 1987 | 0.011 | Ha | 0.05 | 0.014 | < 0.0001 | + | 28.65 | 29.11 |
| T anomaly | 1910-2017 | 2442 | 1976 | < 0.0001 | Ha | 0.56 | 0.019 | < 0.0001 | + | -0.10 | 0.523 |
| Monthly precipitation | 1948-2017 | 11873 | 1970 | 0.0362 | H0 | 0.069 | -0.12 | 0.006 | - | 138.67 | 130.03 |
| Precipitation anomaly | 1948-2017 | 586 | 1970 | 0.0001 | Ha | 0.25 | -1.622 | 0.003 | - | 55.75 | -29.14 |
| Geopotential height | 1948-2017 | 86077 | 1976 | < 0.0001 | Ha | 0.394 | 0.31 | < 0.0001 | + | 88.79 | 105.37 |
| Relative humidity | 1948-2017 | 24125 | 1977 | 0.0004 | Ha | 0.144 | -0.035 | < 0.0001 | - | 44.3 | 42.13 |
| Specific humidity | 1900-2017 | 47200 | 1966 | < 0.0001 | Ha | 0.254 | -0.015 | < 0.0001 | - | 13.34 | 12.42 |
| Zonal wind | 1948-2017 | 56785 | 1975 | < 0.0001 | Ha | 0.60 | 0.068 | < 0.0001 | + | -4.4 | -1.35 |
| AET | 1948-2017 | 36200 | 1995 | < 0.0001 | Ha | 0.45 | 1.2 | < 0.0001 | + | -2.7 | 4.7 |

+ Positive trend, - Negative trend, t change point, Ha- Heterogeneous series, Ho- homogeneous series

**Supplementary Table 2**: Estimation of abrupt changes in the NRB’s hydroclimate based on single and multiple change point (CP) detection methods.

|  | **BCP/MCP/CPM** | | **PELT** | **SCFM** | **BIC** | **DP** |
| --- | --- | --- | --- | --- | --- | --- |
| **Variable** | CP | Probability | CP | CP (p-value <0.005) | CP | CP |
| Temperature | 1975,**1976**, 2001 | 0.84, **0.96**, 0.84 | **1976** | **1975, 1978,** 2001 | **1978**, 2001 | **1976, 1978** |
| Precipitation | 1965, **1971**, 1984 | 0.41, **0.46**, 0.43 | **1971** | **1970**, 1984 | **1970**,1975, 1984 | **1970**, 1975 |
| Relative Humidity | 1970, **1977,** 1978 | 0.85, **0.92**, 0.78 | **1977** | 1970, **1976**, 1993 | **1977**, 1993 | **1977** |
| Wind speed | **1975**, 1983 | **0.89**, 0.64 | **1975** | **1975**, 1983 | **1975**, 1983 | **1975** |
| Specific humidity | **1966**, 1973 | **0.96**, 0.95 | **1966** | **1966** | **1966** | **1966** |
| GPH | 1973, **1976**, 1977 | 0.95, **0.98**, 0.85 | **1976** | **1977**, 1998 | **1977** | **1977** |
| AET | 1976**, 1995** | 0.56, **0.73** | **1995** | **1995** | **1995** | **1995** |

In supplementary Table 2, shifts in the surface temperature data, act in an “up, up, up” warming pattern and evenly placed in the dataset at times = 1975, 1976, and 2001. The SCFM method also identified a three change points but in 1975, 1978, and 2001. In contrast, BIC and DP methods identified only two change points in 1978 and 2001, and only change point was detected by the PELT method in 1976. By visual inspection of the NRB’s surface temperature anomalies dataset, we can clearly identify two distinct warming periods, one beginning around 1970s and the other during the 2000s. However, the detected change point in 1976 have the highest posterior probability of being a true change point, which agree with the identified location of the change point by Pettitt test. The same procedures were repeated for NRB’s precipitation, RH, wind speed, specific humidity, GPH, and AET. In comparison, MCP method becomes increasingly time consuming as the size of the dataset increases, and PELT method identified the exact location of the change point in NRB’s hydroclimate (supplementary Table 1). According to the highest posterior probability of BCP, PELT, visual inspection, and Pettitt test, we can clearly say that there is a change point in NRB’s precipitation, RH, wind speed, specific humidity, GPH, and AET are in 1970, 1977, 1975, 1966, 1976, and 1995, respectively.


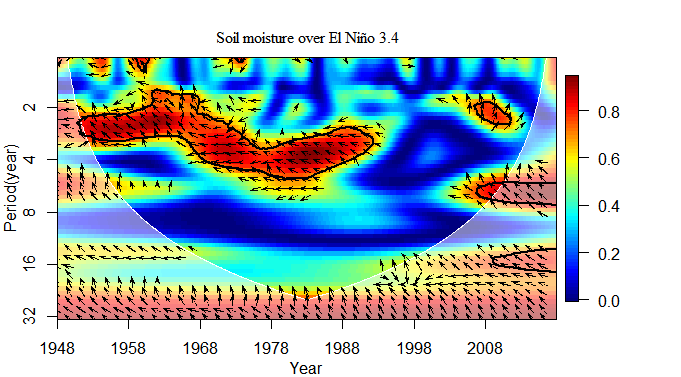


**a**


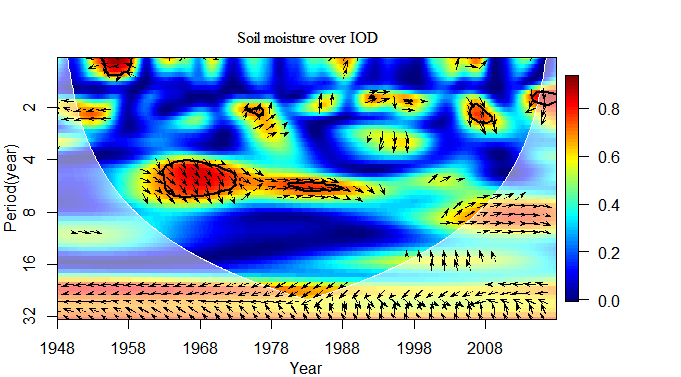


**b**

**Supplementary Fig. 2.** Influence of El Niño 3.4 and IOD on NRB soil moisture content (SMC). WTC between SMC and El Niño 3.4 (a), IOD and SMC (b), SMC time series, where Mb is the long-term average before change point, and Ma the long-term average after change point (c). SMC are computed over 20-years moving periods and then correlated with El Niño 3.4 and IOD amplitudes (d), using ERA-Interim, FLDAS and GLADS soil moisture datasets. The correlation coefficients (at 0.05 significant level) in the top right. The slope is the linear trend estimated and the p-value is the trend significance based on the Mann–Kendall test. In a, the WTC between El Niño 3.4 and NRB’s SMC shows an anti-phase relationship with statistically significant coherence at 1-2, 2-4-year bands over 1960-1998, 2-4-year band over 2000-2017, and an interdecadal, 16-32-year band. In b, IOD and SMC are in-phase, interannual relationships over 1948-2017, but anti-phase relationships at larger timescales. A statistically significant change point of 1979 was detected in SMC which show significant negative trends of 0.84 mm/decade over 1979-2017. These negative trend in SMC is attributed to significantly lower amount of precipitation and higher AET associated with intensive ENSO and WTIO activities over NRB in the same period, as shown by the significant negative (positive) correlation between El Niño 3.4 (IOD) amplitude and SMC (ρ = - 0.61 for Niño 3.4 and 0.52 for IOD) (d).

**b**

**a**

TWS and IOD

Niño 3.4

TWS and El Niño 3.4


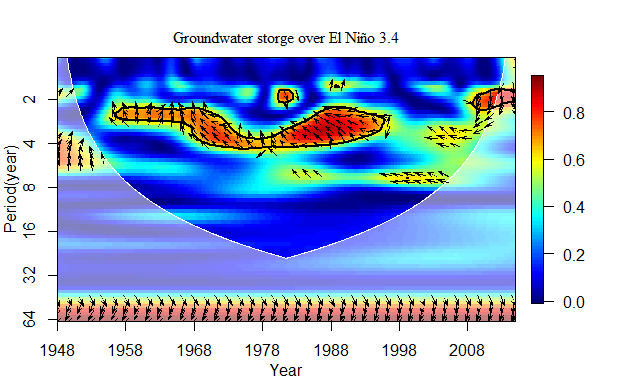

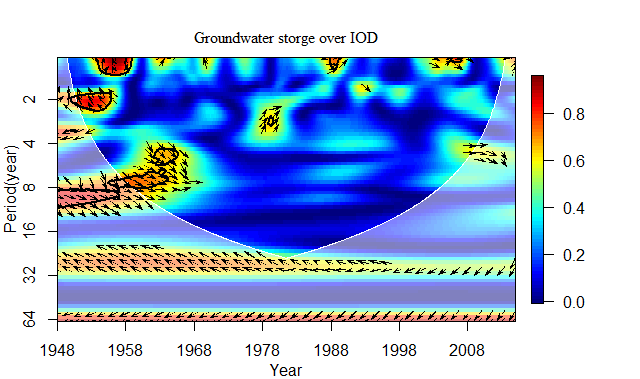


**Supplementary Fig. 3**. Same as in supplementary Fig. 2 but for total water storage (TWS). TWS are area averaged TWS storage from computed from water balance model, GLADS-CLSM025 between 1948-2018, and the Gravity Recovery and Climate Experiment (GRACE) between 2003 and 2017. A downward trend of -1.44 mm/decade in monthly TWS was observed after 1979 (c). This trend could be attributed to a significantly lower amount of precipitation, higher AET and lower SMC after 1979. In d, a statistically significant negative correlation can be seen between El Niño 3.4 amplitude and TWS (ρ = -0.72), however a moderate positive correlation with IOD amplitude was detected (ρ = 0.43).

**b**

**c**

**a**

Surface runoff (mm)

Potential evaporation (mm)

Air temperature (^o^C)

Stream function (m^2^/s)

Soil moisture (mm)

RH (%)

**f**

**e**

**d**

**Supplementary Fig.** 4: Composite maps of (a) air temperature (^0^C), (b) relative humidity (%), (c) soil moisture (mm), (d) surface runoff (mm), (e) potential evaporation (mm), and (f) 300-mb stream function derived from data of 1985-2017 minus data of 1948-1984. In a, Ts shows an increase of 0.16-0.4 C^o^/decade over the NRB, with highest increase observed in Sudan and Egypt. This finding agrees well with the earlier results from trend analysis. In b, RH has decreased by 1-5%/decade after 1985, with the largest decrease in Ethiopia, Uganda, and Sudan where warming has also been the worst. Significant evaporation losses recently observed in water bodies of many riparian countries of NRB are related to strong wind stress (e), where areas with high PET correspond to higher Ts and GPH but lower RH. The surface soil moisture a decreasing trend of 16-45 mm/decade between 1985 and 2017 (c). In d, a slight decrease in surface runoff can also be seen in Ethiopia, Uganda, and Sudan, while there is no change over Egypt. In f, the lower atmosphere stream function at 0.8458 sigma level, which depicts the rotational part of the flow (the flow is along the contours), indicates that main waves emanating from northern towards southern parts of NRB, have shifted further south over the two periods, from 1948-1984 to 1985-2017. This long-term southward shift in the stream function over NRB would have also contributed towards the long-term drying of NRB, as part of the multiple changes, e.g., changes in Ts, GPH, RH, soil moisture, surface runoff, PET and ENSO. The maps were generated with NCAR Command Language (NCL) Version 6.2.1 (http://www.ncl.ucar.edu/).

**a**

**b**

**d**

Precipitation (mm)

Specific humidity (g/kg)

Meridional wind (m/s)

Scalar wind (m/s)

**c**

Zonal wind (m/s)

GPH (m)

**e**

**f**

**Supplementary Fig.** 5: Composite maps of (a) Precipitation, (b) Specific humidity, (c) Scalar wind, (d) Meridional wind, (e) Zonal wind, (f) GPH for 1985 to 2017 minus 1948 to 1984. In a, the composite mean map of precipitation shows a drop in daily precipitation in the NRB except for Uganda which had an increase of 1.5-3.5 mm/day between 1985 and 2017. In c, the increase in scalar wind was maximum in Uganda, Sudan, and northwestern regions of Ethiopia with an average decline of 0.2-0.8 m between 1985 and 2017 (c). Meridional and Zonal wind also showed similar increase in these regions (d-e). In f, GPH shows 5-15 m increase in the NRB. The highest increase was in Egypt and Sudan (downstream) with an average increase of 3-5 m/decade. Upstream riparian of the basin such as Ethiopia had an increase of 1.5-3.75 m/decade. These results revealed that GPH become thicker in the last few decades as the temperature increase. The maps were generated with NCAR Command Language (NCL) Version 6.2.1 (<http://www.ncl.ucar.edu/>).

**Supplementary Fig. 6.** IOD-ENSO relationship over the NRB basin: (a) wavelet coherence (WTC) between El Nino 3.4 and IOD index, and (b) DCCA between El Niño 3.4 and IOD amplitudes. El Niño 3.4 and IOD amplitude computed as the SD of El Niño 3.4 and IOD over 30-year window from 1913 to 2017. In a, the arrows show the phase difference: right-pointing arrows indicates that the two-time series are in phase signals, while left-pointing arrows represents anti-phase signals and mean that one time series leads (lags) the other by 90^o^. In a, shows the IOD’s power spectrum and significant coherence with ENSO at 1-2, 2-4, 4-8, 14-16, and 32-64 year time scales, which demonstrate a strong coupling between IOD and ENSO, for both exhibited similar change patterns, e.g., positive IOD becomes more intensive as the amplitude of El Niño increases. In 1-2- and 2-4-year bands, ENSO and IOD are almost in phase, but are out of phase by about a year in other periodicities. In b, there is a significant positive correlation between El Niño and IOD amplitudes (ρ = 0.32). This positive correlation indicates that positive IOD occur simultaneously with El Niño. In b, IOD seems to have an independent mode of variability from El Niño. For instance, a strong positive IOD event (shown by a yellow mark in b) occurred in 1961 under a neutral ENSO conditions (blue colour in b). It seems that the ENSO–IOD teleconnection has strengthened since the 1970s partly because of the recent enhancement of the Walker circulation, resulting in significant positive correlation between El Niño and IOD amplitudes.


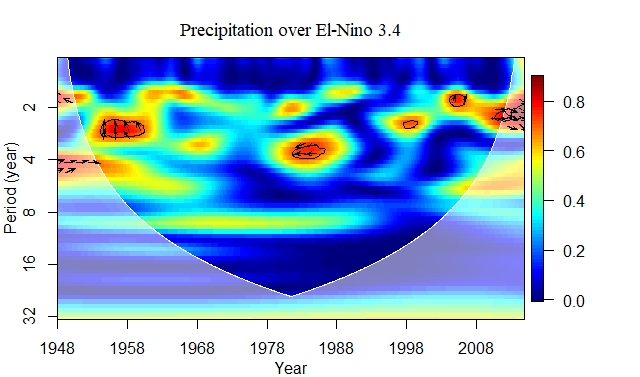

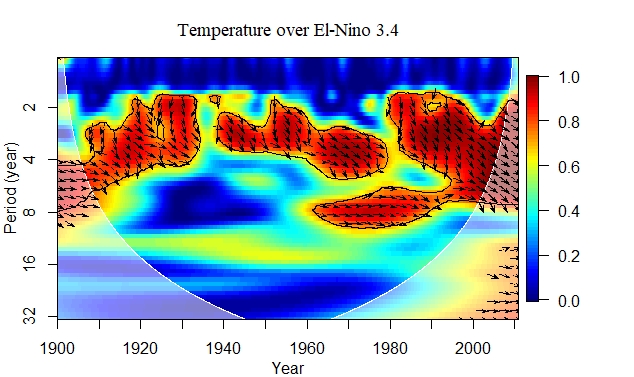

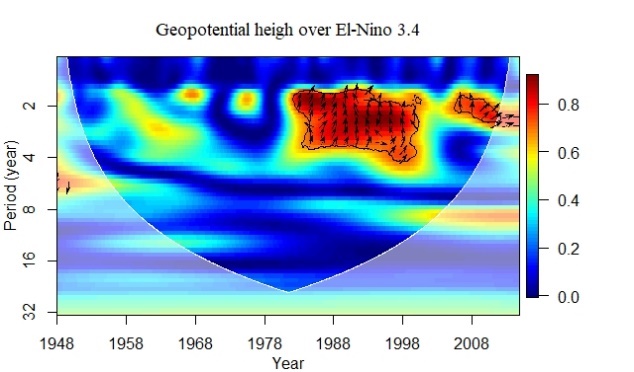

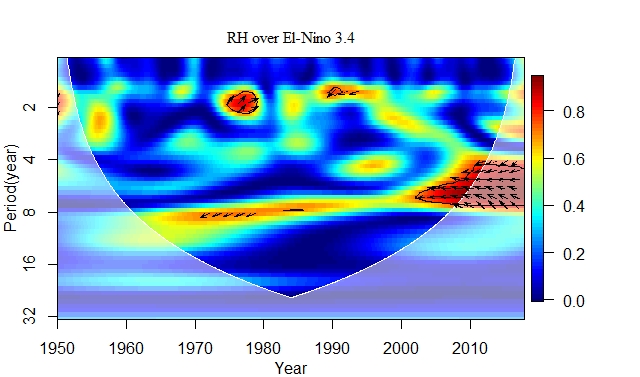


**d**

**c**

**b**

**a**


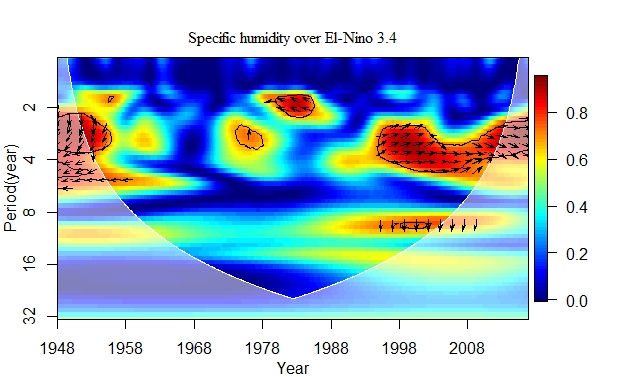

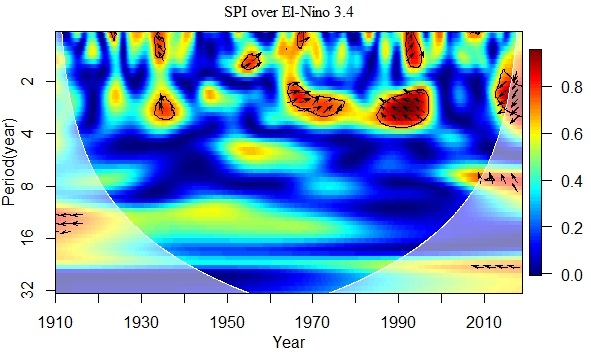


**f**

**e**


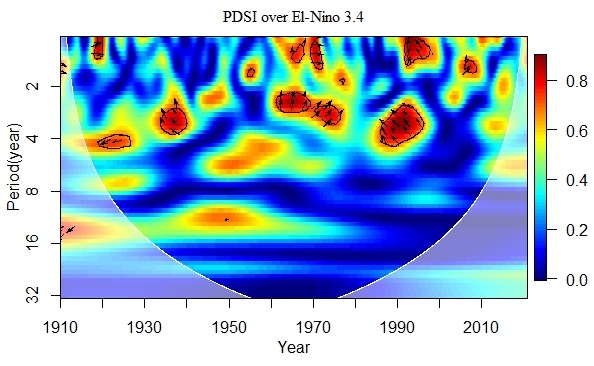

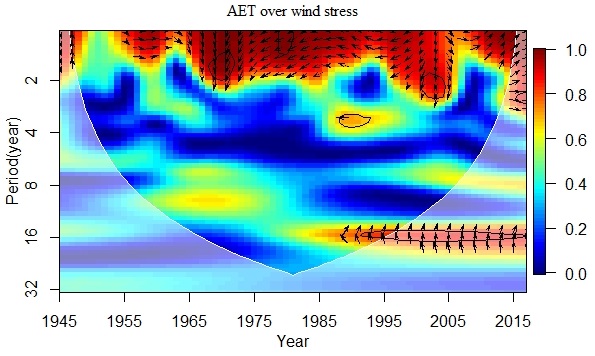


**h**

**g**


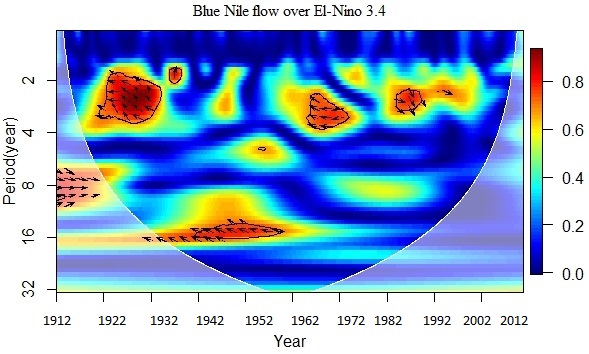

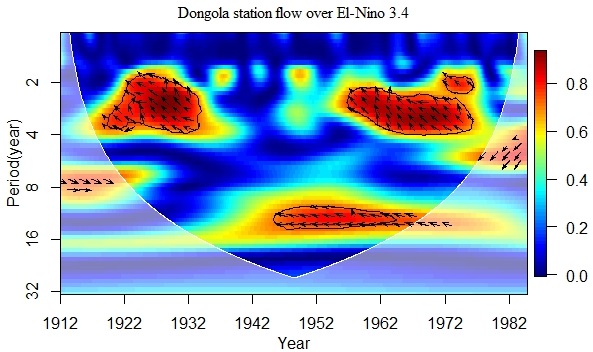


**k**

**j**

**i**


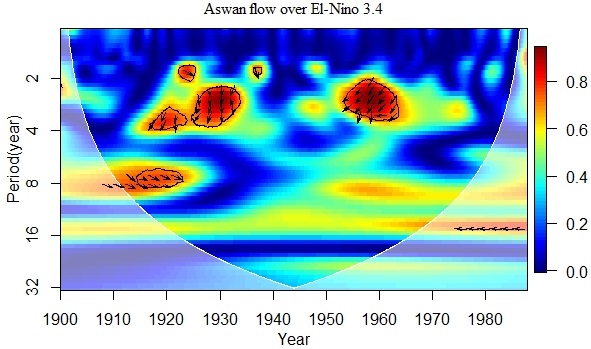


**Supplementary Fig.** 7: WTC between El Nino 3.4 and (a) precipitation, (b) Ts, (c) GPH, (d) RH, (e) specific humidity, (f) SPI, (g) sc-PDSI, (h) AET and wind stress, (i) Blue Nile flow, (j) Dongola station flow, and (k) Aswan station flow. In a, precipitation exhibits coherent spatial patterns with respect to El Niño, as WTC revealed a statistically significant coherency at 2-4 yr band in 1960s and 1970s and indicates anti-phase relationships between precipitation and El Nino after 1970s. The WTC between El Nino and Ts (GPH) in b (c), show statistically significant power at 2–4-yr bands in 1920 to 1940, 1950 to 1970, and 1975 to 2012 over NRB. This observed strong in-phase relationship between El Nino and Ts and GPH after 1970 indicates that El Nino leads the warming trend in the NRB. In d, there is anti-phase relationship between El Nino 3.4 and RH. In f and g, El Nino had significant coherency with SPI and sc-PDSI after 1970s compared to drought conditions in the earlier years. These results suggest that a higher El Nino index usually corresponds to a lower SPI and sc-PDSI value (more severe drought) and explains the more frequent droughts in the NRB after 1970. WTC plots between wind stress and AET also shows that wind stress and AET were in phase after 1975 (h), which indicate that the increase in wind stress after 1970s drives the increase in actual evapotranspiration in the NRB. WTC between El Nino and the Blue Nile flow (i), shows that the anti-phase signals between El Nino and the Blue Nile flow clearly dominate the entire period between 1912 and 2012 except for a small in phase signals between 1982 and 1990, implying that El Nino has large influence on the Blue Nile flow. In j-k, El Nino had similar effects on the NRB’s flow at Dongola and Aswan station.


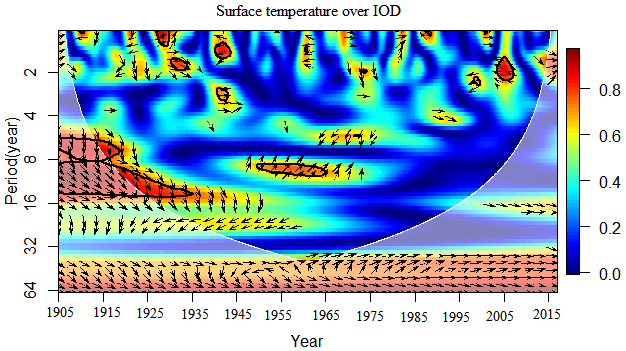


**a**


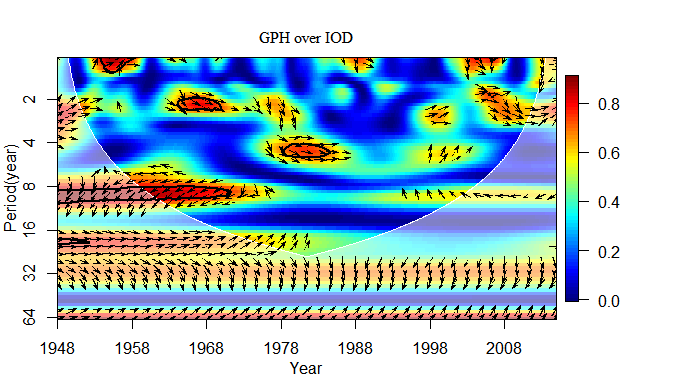


**b**


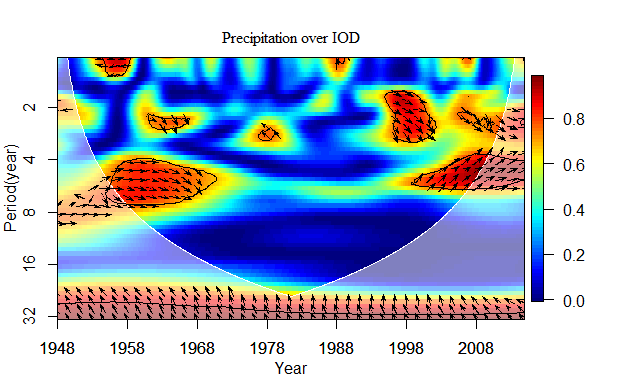


**c**


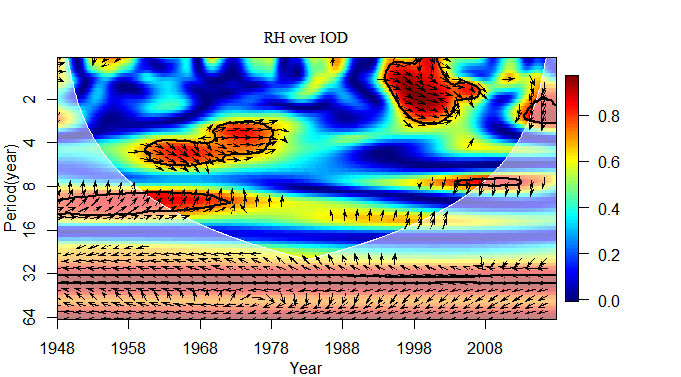


**d**


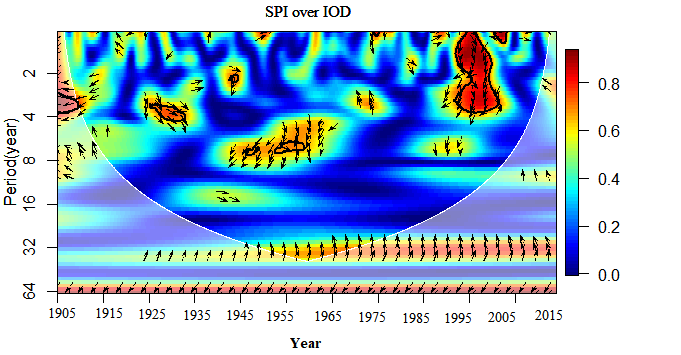


**e**


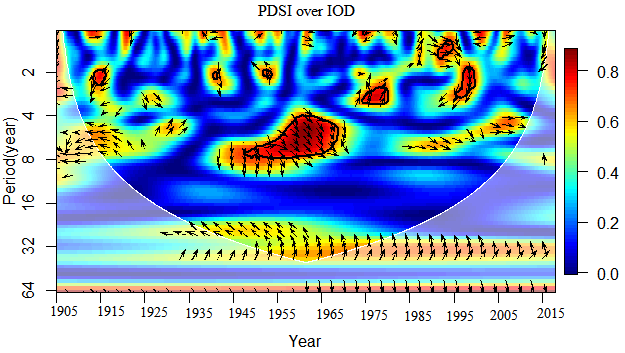


**f**

**Supplementary Fig. 8.** Wavelet coherence between IOD and (a) surface temperature, (b) GPH, (c) precipitation, (d) relative humidity, (e) SPI, and (f) PDSI. In a, the WTC between IOD and surface temperature (T_s_) shows statistically significant, interannual (1-4yr) in-phase and anti-phase relationships over 1905 and 2018, revealing the interannual response of T_s_ and GPH to the variability of IOD in the last 100 years. At inter-decadal time scale, IOD and Ts show only significant, in-phase coherence at 8–16, 16-32, 32-64-year bands over 1975-2018. In b, IOD and GPH were continuously in phase with significant coherent spatial pattern at 2-4- and 4-8-years band after 1970s. On the other hand, WTC between precipitation and IOD shows in phase relationship with significant coherent spatial pattern at 2-4- and 4-8-year band after 1990, and an anti-phase relationship at 32-year band between 1948 and 2018 (c). This strong anti-phase relationship shows that IOD primarily influenced NRB’s precipitation at inter-decadal timescales. There is a strong in-phase relationship between IOD and relative humidity at 2-4 yr band over 1998-2008, and at 4-8 and 8-16-year bands over 1960-1980, and an anti-phase relationship in the 32-64-year band (d). In e-f, the WTC between IOD and SPI and sc-PDSI are statistically significant at 2–4, 4-8, and 32-64-year bands, respectively. Before 1970s, IOD was in-phase with both sc-PDSI and SPI, but after 1970s their relationships became predominantly anti-phase, which means that stronger positive IOD episodes after 1970s has led to lower SPI and sc-PDSI, resulting in more severe droughts in NRB after 1970s (e-f).

**Supplementary Fig. 9.** WTC between NRB’s AET and El Niño (a), AET and IOD (b), AET and zonal and meridional wind stresses (c, d). DCCA between NRB AET and El Niño 3.4 and IOD amplitudes (e), and wind stresses (f). AET, meridional and zonal wind stresses computed over 20-year running periods from 1950 to 2017, El Niño 3.4 and IOD amplitude are the SD of El Niño and IOD indexes over 20-year windows from 1950 to 2017 using the ERSST data sets. The numbers in the top right are the cross-correlation coefficient at the 5% level. In a-b, the WTC plots between AET and El Niño and IOD show in-phase, statistically significant coherent relationship at 2-4 and 8-14-years band after 1970s, which peaked at the 14–16-year time scale after 2000s. In c-d, there is also a strong WTC between zonal and meridional wind stresses and AET at 1-2 years bands. Their recurrent in-phase and anti-phase relationships at 1-2 years bands demonstrate the effect of positive (negative) wind stress anomalies when El Niño was active. DCCA also shows a significant positive correlation between AET and El Niño 3.4 (ρ =0.93) and meridional wind stresses (ρ = 0.62) in contrast to negative correlation between AET and IOD (ρ = -0.47) and zonal wind stresses (ρ = -0.37) (e, f), which shows that El Niño and meridional wind affect the AET of NRB.


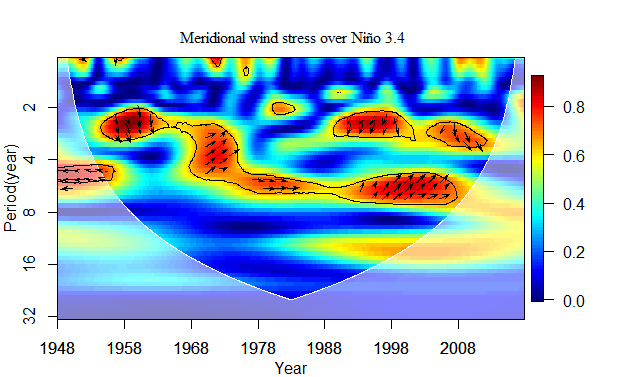


**a**


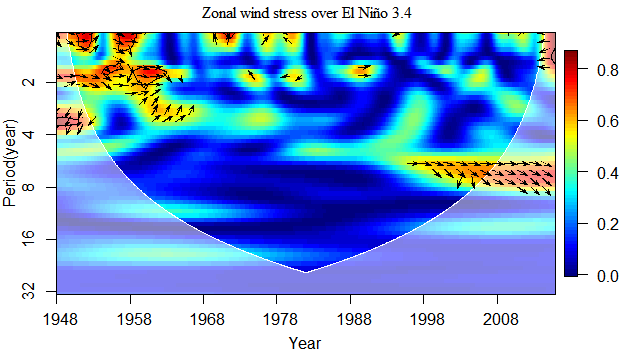


**b**


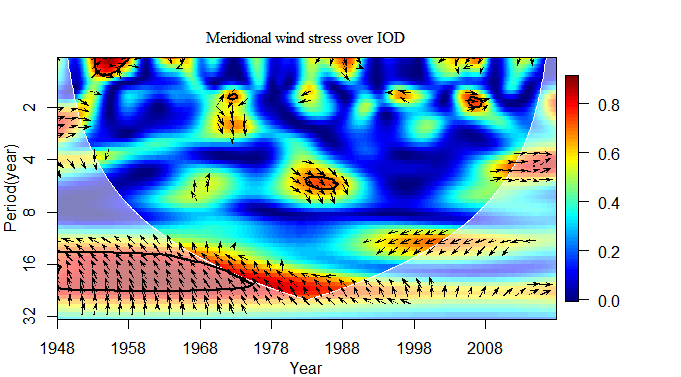


**c**


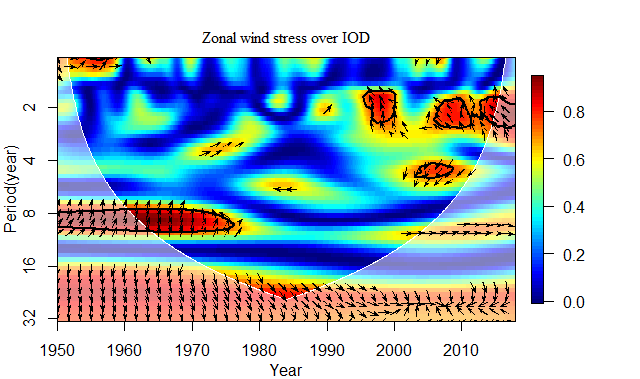


**d**

**Supplementary Fig. 10.** WTC between El Nino, IOD and wind stresses: WTC between El Niño and meridional and zonal wind stresses (a, b), IOD and meridional and zonal wind stresses (c, d). In a and b, there is a statistically significant in-phase relationship between meridional wind stress and El Niño at 2-4 and 4-8 year band was detected over 1960-2017, and in-phase relationship between zonal wind stress and El Niño at the 4-8-year band 1988 and 2017, while IOD, meridional and zonal wind stresses showed in-phase coherence after 2000s (c, d). It seems both increasing meridional wind stress anomalies and stronger El Niño episodes have contributed to changes in the heat content of NRB, rising Ts and AET.

**Supplementary** Table 3: Change points and trend analysis of sc-PDSI, temperature and precipitation in each riparian country

|  |  | **Pettitt test** | | | | **Mann–Kendall test** | | | |  |  |
| --- | --- | --- | --- | --- | --- | --- | --- | --- | --- | --- | --- |
| **Ethiopia subbasin** | Years | K | t | P | trend | Tau | Sen's slope | P | trend | Mb | Ma |
| PDSI time series | 1960-2013 | 60763 | 1983 | < 0.0001 | Ha | 0.35 | -0.058 | < 0.0001 | - | 0.338 | -1.36 |
| Temperature time series | 1960-2014 | 29866 | 1994 | < 0.0001 | Ha | 0.44 | 0.023 | < 0.0001 | + | 20.934 | 21.6 |
| Temperature anomaly | 1960-2015 | 601 | 1994 | <0.0001 | Ha | 0.653 | 0.028 | <0.0001 | + | -0.23 | 0.424 |
| Precipitation time series | 1960-2015 | 3794 | 1968 | 0.007 | Ha | 0.01 | -0.014 | 0.021 | - | 89.156 | 80.94 |
| Precipitation anomaly | 1960-2015 | 200 | 1977 | 0.02 | Ha | 0.073 | **-1.801** | 0.04 | - | 40.15 | -30.2 |
| **Uganda subbasin** |  |  |  |  |  |  |  |  |  |  |  |
| PDSI time series | 1960-2013 | 50079 | 1979 | < 0.0001 | Ha | 0.321 | -0.058 | < 0.0001 | - | 0.379 | -1.314 |
| Temperature time series | 1960-2014 | 55944 | 1994 | < 0.0001 | Ha | 0.485 | 0.033 | < 0.0001 | + | 22.255 | 23.42 |
| Temperature anomaly | 1960-2013 | 645 | 1994 | <0.0001 | Ha | 0.694 | 0.033 | 0.03 | + | -0.41 | 0.756 |
| Precipitation time series | 1960-2015 | 6800 | N | 0.687 | Ho | 0.03 | -0.113 | 0.279 | - | 104.704 | |
| Precipitation anomaly | 1960-2013 | 197 | 1978 | 0.025 | Ha | 0.073 | **-0.864** | 0.003 | - | 29.69 | -37.9 |
| **Kenya subbasin** |  |  |  |  |  |  |  |  |  |  |  |
| PDSI time series | 1960-2013 | 45349 | **1991** | < 0.0001 | Ha | 0.28 | -0.056 | < 0.0001 | - | 0.126 | -1.424 |
| Temperature time series | 1960-2014 | 42974 | **1994** | < 0.0001 | Ha | 0.444 | 0.026 | < 0.0001 | + | 21.258 | 22.16 |
| Temperature anomaly | 1960-2014 | 634 | **1994** | <0.0001 | Ha | 0.611 | 0.028 | 0.002 | + | -0.317 | 0.584 |
| Precipitation time series | 1960-2015 | 6032 | **N** | 0.419 | Ho | 0.023 | -0.017 | 0.401 | - | 94.764 | |
| Precipitation anomaly | 1960-2014 | 171 | **1968** | 0.0.3 | Ha | 0.11 | **-1.801** | 0.05 | - | 110.07 | -52.4 |
| **Tanzania subbasin** |  |  |  |  |  |  |  |  |  |  | |
| PDSI time series | 1960-2013 | 50493 | **1991** | < 0.0001 | Ha | 0.263 | -0.045 | < 0.0001 | - | 0.148 | -1.314 |
| Temperature time series | 1960-2014 | 33073 | **1986** | < 0.0001 | Ha | 0.433 | 0.021 | < 0.0001 | + | 22.295 | 22.96 |
| Temperature anomaly | 1960-2014 | 659 | **1986** | <0.0001 | Ha | 0.639 | 0.022 | < 0.0001 | + | -0.325 | 0.325 |
| Precipitation time series | 1960-2015 | 7144 | **N** | 0.872 | Ho | 0.064 | -0.103 | 0.019 | - | 85.492 | |
| Precipitation anomaly | 1960-2015 | 236 | **1991** | 0.049 | Ha | 0.215 | **-2.418** | 0.028 | - | -0.318 | -80.58 |
| **Sudan subbasin** |  |  |  |  |  |  |  |  |  |  |  |
| PDSI time series | 1960-2013 | 35944 | **1968** | < 0.0001 | Ha | 0.124 | -0.017 | < 0.0001 | - | 0.171 | -1.49 |
| Temperature time series | 1960-2014 | 23117 | **1979** | < 0.0001 | Ha | 0.439 | 0.039 | < 0.0001 | + | 27.198 | 28.33 |
| Temperature anomaly | 1960-2014 | 603 | **1978** | <0.0001 | Ha | 0.677 | 0.039 | <0.0001 | + | -0.735 | 0.399 |
| Precipitation time series | 1960-2015 | 4754 | **N** | 0.129 | Ho | 0.013 | -0.01 | 0.647 | - | 30.154 | |
| Precipitation anomaly | 1960-2015 | 241 | **1968** | 0.01 | Ha | 0.124 | **-0.757** | 0.11 | - | 42.77 | -13.03 |
| **Congo ‘Zaire’ subbasin** |  |  |  |  |  |  |  |  |  |  |  |
| PDSI time series | 1960-2013 | 15801 | **1963** | 0.001 | Ha | 0.04 | -0.003 | 0.104 | - | 2.406 | 0.003 |
| Temperature time series | 1960-2014 | 16342 | **1985** | 0.005 | Ha | 0.197 | 0.007 | < 0.0001 | + | 24.429 | 24.78 |
| Temperature anomaly | 1960-2014 | 501 | **1982** | <0.0001 | Ha | 0.318 | 0.007 | 0.001 | + | -0.194 | 0.144 |
| Precipitation time series | 1960-2015 | 5165 | **N** | 0.248 | Ho | 0.040 | -0.141 | 0.147 | - | 131.425 | |
| Precipitation anomaly | 1960-2015 | 42.3 | **1970** | 0.049 | Ha | 0.163 | **-1.905** | 0.01 | - | 77.43 | -20.93 |
| **Burundi subbasin** |  |  |  |  |  |  |  |  |  |  |  |
| PDSI time series | 1960-2013 | 18044 | **1990** | < 0.0001 | Ha | 0.054 | -0.004 | 0.049 | - | 0.122 | -0.404 |
| Temperature time series | 1960-2014 | 54455 | **1994** | < 0.0001 | Ha | 0.39 | 0.02 | < 0.0001 | + | 20.129 | 20.9 |
| Temperature anomaly | 1960-2014 | 656 | 1989 | <0.0001 | Ha | 0.564 | 0.021 | 0.02 | + | -0.31 | 0.388 |
| Precipitation time series | 1960-2015 | 5518 | **N** | 0.278 | Ho | 0.009 | -0.072 | 0.013 | - | 102.6 | |
| Precipitation anomaly | 1960-2014 | 280 | 1989 | 0.04 | Ha | 0.389 | -1.917 | 0.008 | - | 27.53 | -46.48 |
| **Rwanda subbasin** |  |  |  |  |  |  |  |  |  |  |  |
| PDSI time series | 1960-2013 | 18435 | 1969 | < 0.0001 | Ha | 0.023 | -0.04 | 0.039 | - | 0.836 | -0.227 |
| Temperature time series | 1960-2014 | 65428 | 1994 | < 0.0001 | Ha | 0.399 | 0.021 | < 0.0001 | + | 18.729 | 19.59 |
| Temperature anomaly | 1960-2014 | 663 | 1990 | <0.0001 | Ha | 0.543 | 0.022 | < 0.0001 | + | -0.326 | 0.44 |
| Precipitation time series | 1960-2015 | 5192 | N | 0.198 | Ho | 0.021 | -0.013 | 0.440 | - | 99.614 | |
| Precipitation anomaly | 1960-2015 | 254 | 1983 | 0.123 | Ha | 0.126 | -1.558 | 0.10 | - | 34.56 | -35.56 |
| **Eritrea subbasin** |  |  |  |  |  |  |  |  |  |  |  |
| PDSI time series | 1960-2013 | 54130 | **2001** | < 0.0001 | Ha | 0.207 | -0.048 | < 0.0001 | - | 0.201 | -2.72 |
| Temperature time series | 1960-2014 | 24322 | **1979** | < 0.0001 | Ha | 0.431 | 0.03 | < 0.0001 | + | 23.656 | 24.57 |
| Temperature anomaly | 1960-2013 | 579 | **1987** | <0.0001 | Ha | 0.649 | 0.03 | <0.0001 | + | -0.573 | 0311 |
| Precipitation time series | 1960-2015 | 6498 | N | 0.593 | Ho | 0.028 | -0.004 | 0.305 | - | 28.05 | |
| Precipitation anomaly | 1960-2015 | 263 | 1988 | 0.10 | Ha | 0.175 | -1.505 | 0.06 | - | 27.34 | -37.02 |

**Supplementary** Table 4: Change point and trend analysis of metrological drought in the NRB

|  |  | **Pettitt test** | | | | **Mann–Kendall test** | | | |  |  |
| --- | --- | --- | --- | --- | --- | --- | --- | --- | --- | --- | --- |
| Nile river basin | Years | K | t | P | trend | Tau | Sen's slope | P | trend | Mb | Ma |
| 1-month SPI | 1950-2017 | 79564 | 1979 | < 0.0001 | Ha | 0.362 | -0.015 | < 0.0001 | - | 0.223 | -0.225 |
| 3-month SPI | 1950-2017 | 101530 | 1979 | < 0.0001 | Ha | 0.464 | -0.02 | < 0.0001 | - | 0.321 | -0.31 |
| 6-month SPI | 1950-2017 | 117354 | 1979 | < 0.0001 | Ha | 0.548 | -0.023 | < 0.0001 | - | 0.387 | -0.381 |
| 12-month SPI | 1950-2017 | 132794 | 1979 | < 0.0001 | Ha | 0.638 | -0.027 | < 0.0001 | - | 0.451 | -0.852 |


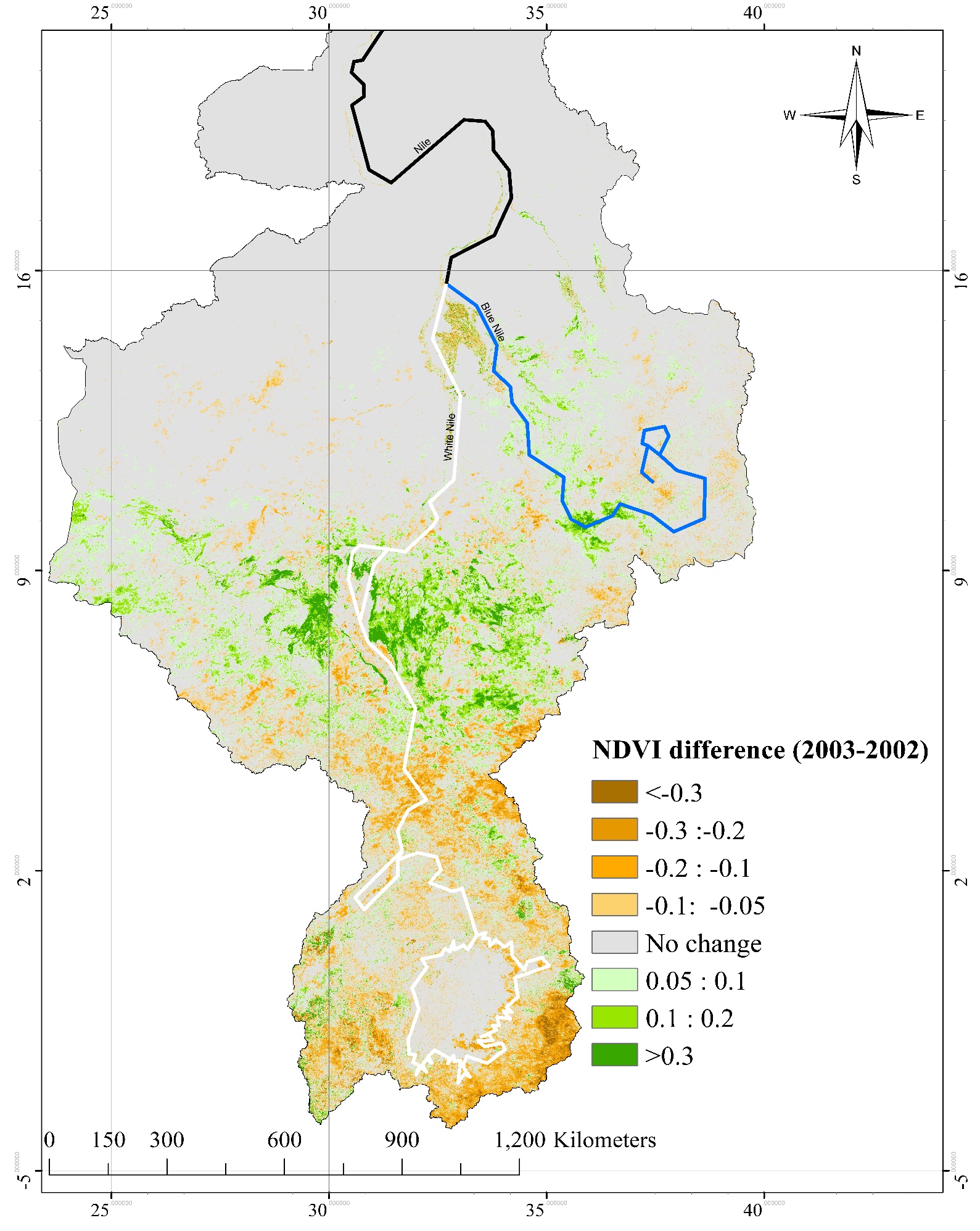

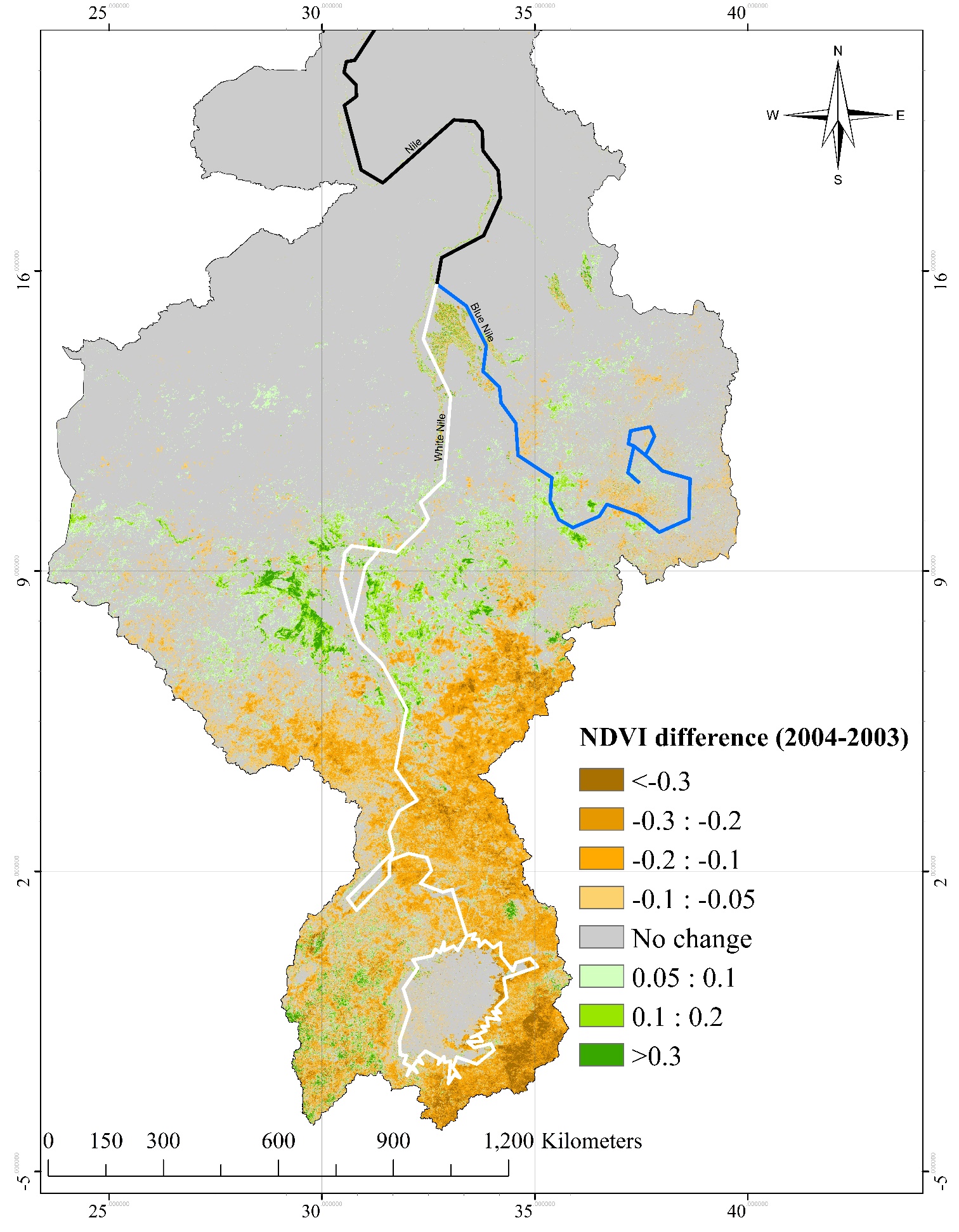

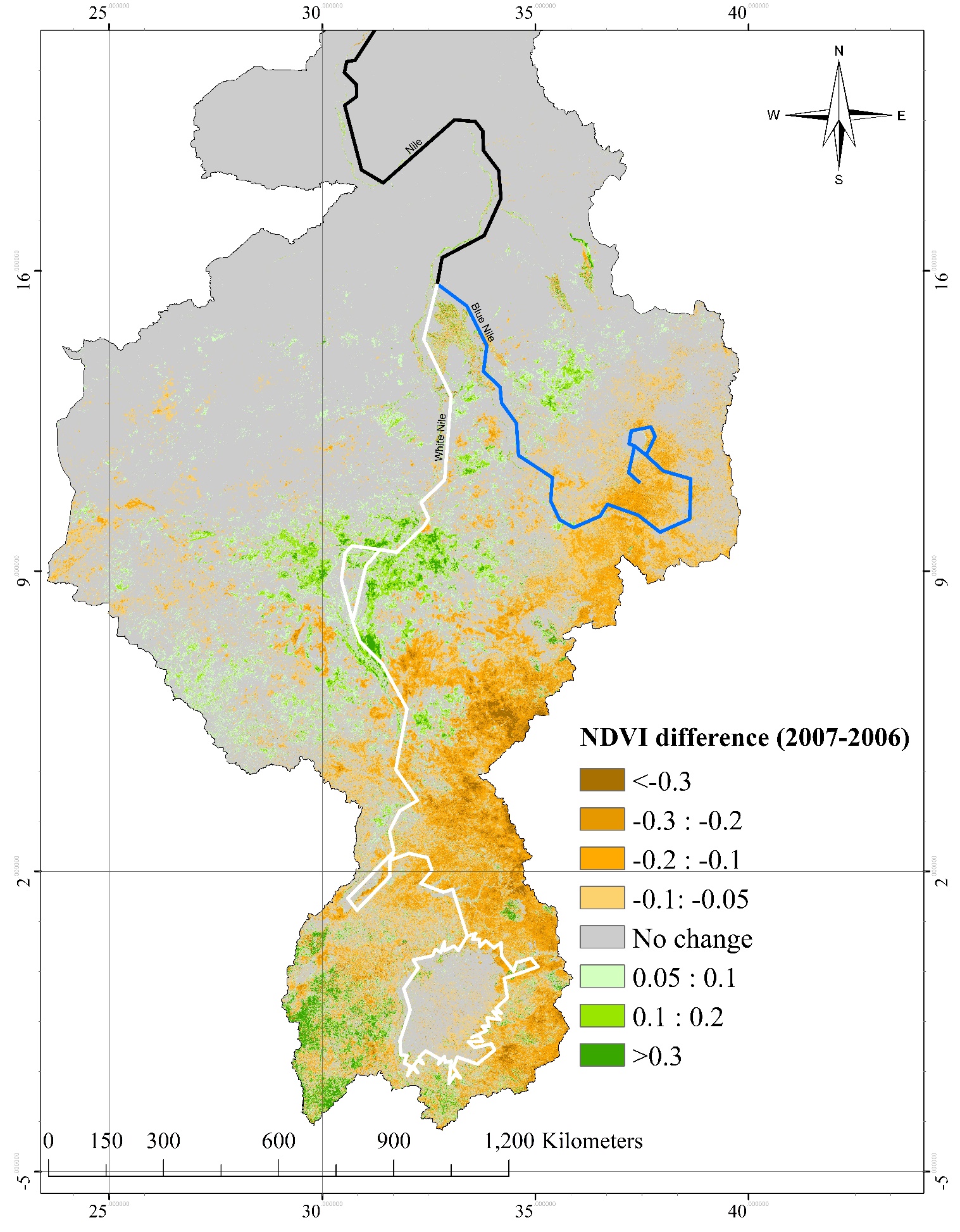

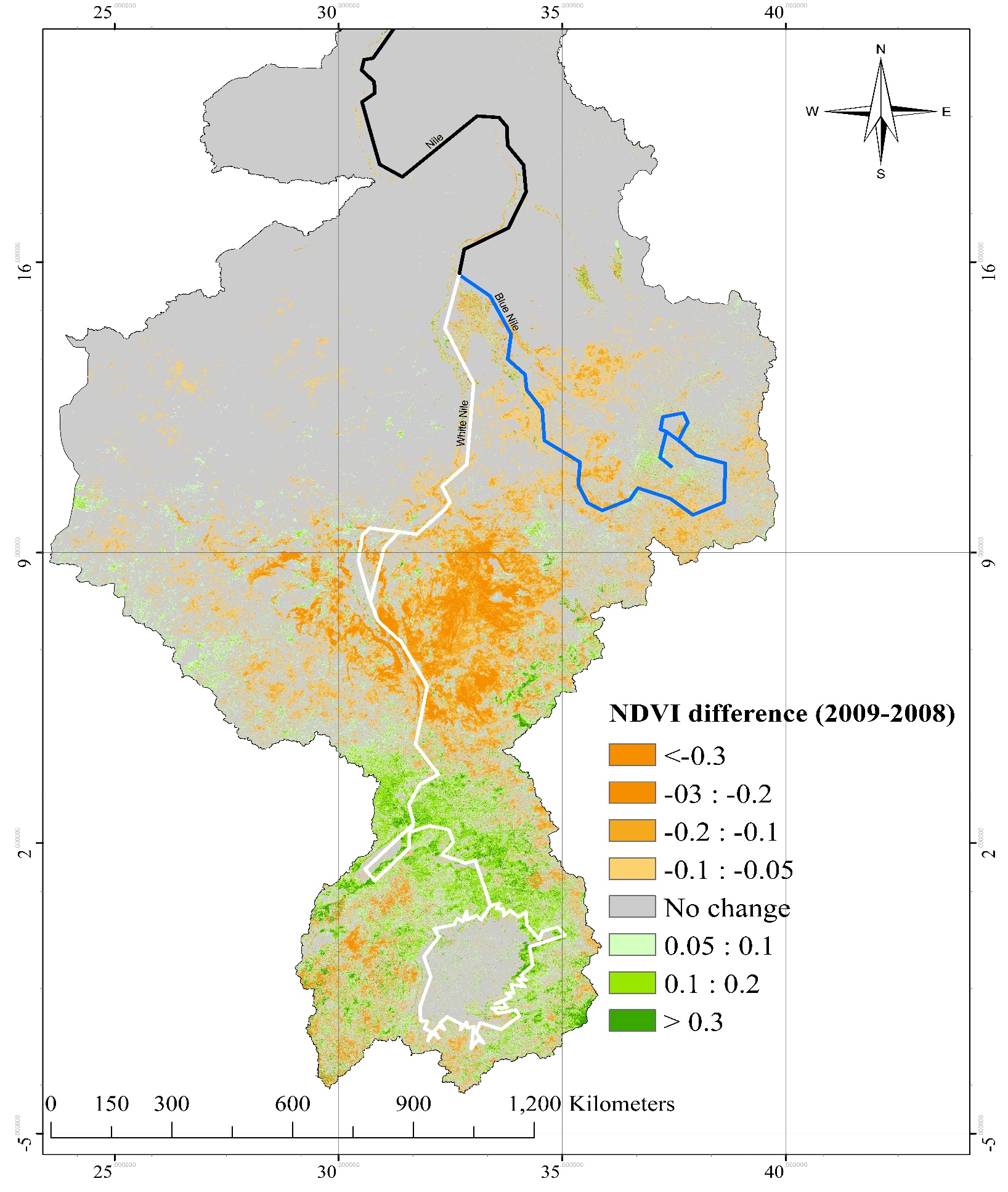

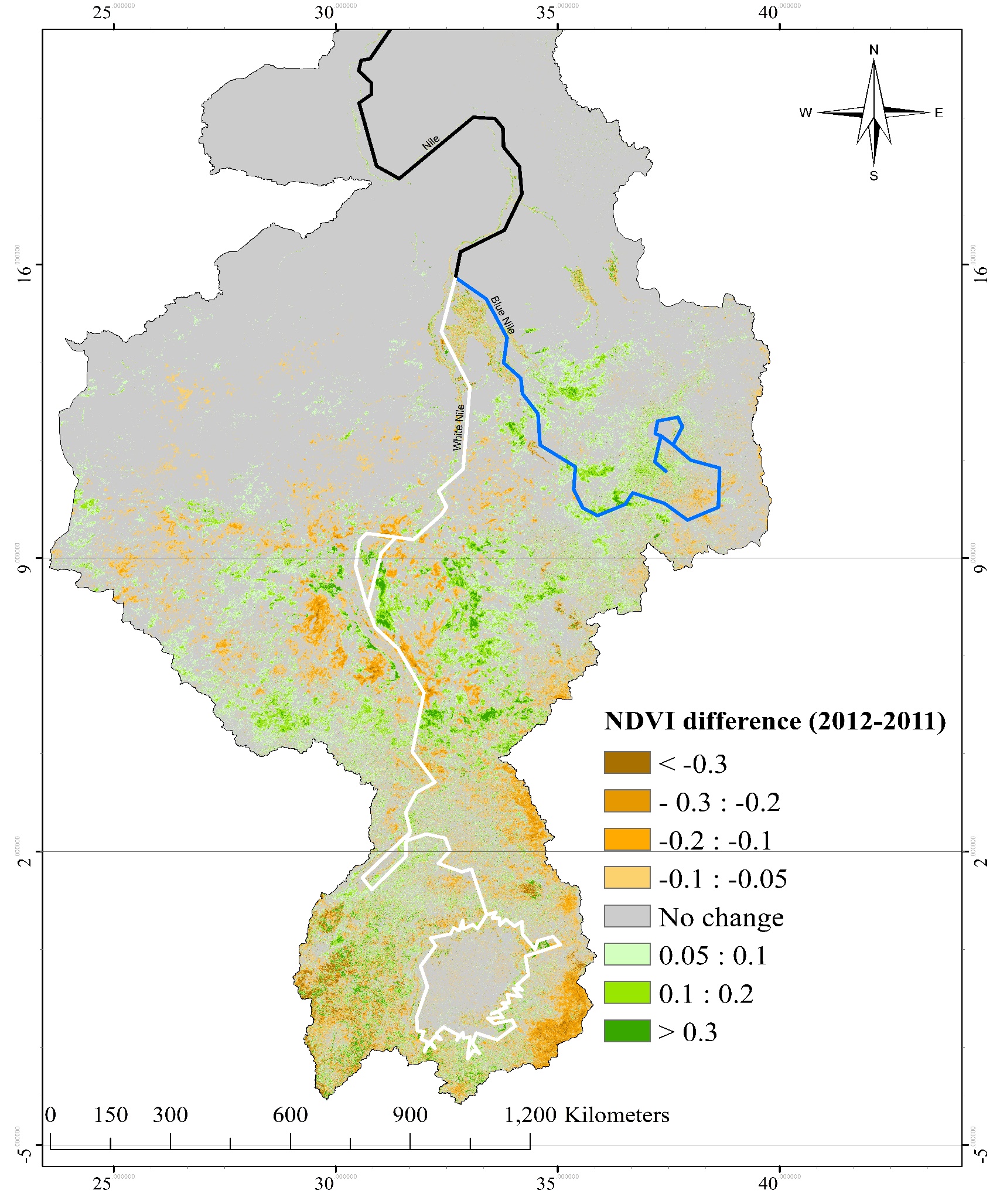

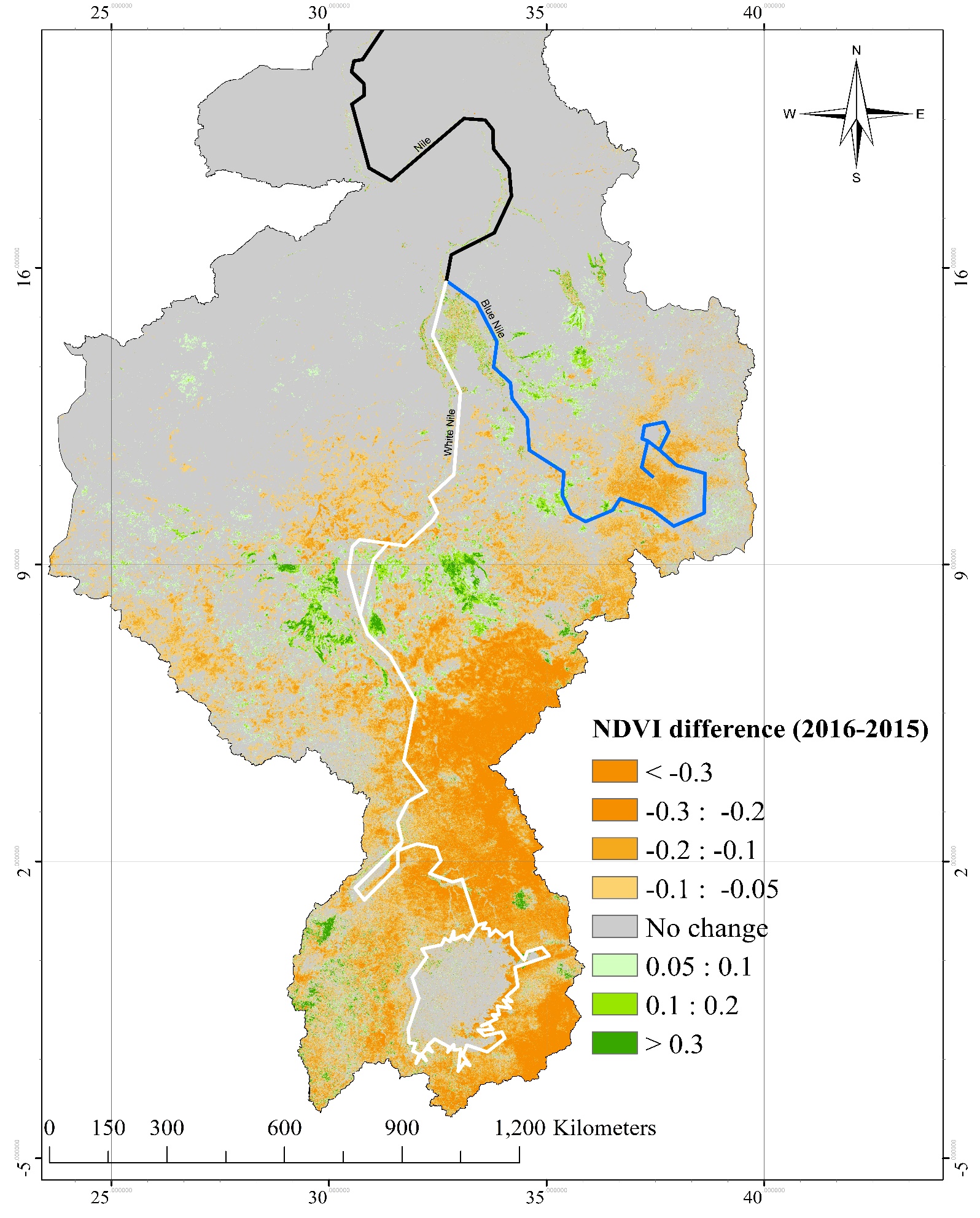


**Supplementary Fig. 11:** Agricultural drought change detection between 2002 and 2016. Fig.14 shows a large variation of droughts in various regions in the NRB. Difference between NDVI values in 2002-2003 revealed a decrease in NDVI values up to 0.3 which indicate large decrease in vegetation cover, with the highest observed decrease in vegetation cover were in Tanzania, Uganda, Rwanda, Burundi, Congo, Ethiopia, and Sudan. The difference between 2004-2003 also showed continuous drought trend with increase in amplitude which cover more areas than the observed drought in 2003, the entire regions of Ethiopia, Eritrea, Kenya, Tanzania, Congo, and Uganda faced severe agriculture droughts in 2004. This result agrees with the observed trend in SPI where 2003 and 2004 were classified as moderate droughts years, it seems like what might be moderate metrological drought can have severe consequences in agricultural drought. Between 2007 and 2006, this trend expanded in Eritrea, Uganda, Rwanda, Burundi, Congo, and Sudan-which also confirm the result obtained from SPI trend analysis in the same year. To confirm the results obtained from agricultural drought analysis, we considered NDVI values in the year 2012 where no drought was captured by SPI index, difference between 2012 and 2011 showed vegetation recovery and an increase in agricultural areas, which in some way confirm our results from the SPI drought analysis. The maps were generated with ArcMap Version 10.1 (http://www.esri.co m/en/arcgis/ arcgis-for-desktop/).

**Supplementary Fig. 12.** Impact of SEIO and WTIO on NRB precipitation and drought. Wavelet coherence between SEIO and WTIO and precipitation (a, b), Wavelet coherence between SEIO and WTIO and PDSI (c, d), WTC between SEIO and WTIO and SPI (e, f). In a-b, there is a more prominent anti-phase relationship between WTIO and NRB’s precipitation anomalies at 2-4, 4-8, and 8-16-year bands after 1970s than the relationship between SEIO and NRB’s precipitation. A stronger anti-phase relationship was also detected between WTIO and SPI and scPDSI throughout most of the period between 1905 and 2018, except between 1950 and 1965 (d-f). This dominant anti-phase relationship between WTIO and NRB’s precipitation at interdecadal timescale (> 32-year) show that NRB’s hydroclimate is more strongly linked to the SST gradient over the Arabian Sea than by IOD and SEIO, such that WTIO exerted a significant negative influence on the NRB precipitation variability (ρ = -0.82).

**Supplementary Fig. 13.** WTIO and SEIO Teleconnections on NRB precipitation and droughts. Field correlation between SEIO and WTIO and NRB precipitation (a), correlation between SEIO and WTIO and NRB SPI (b), and (c) correlation between SEIO and WTIO and NRB PDSI. The result implies that the more frequent occurrences of droughts in NRB are related to increased warming in the western Indian Ocean, as is also evident from the strong negative correlation between SPI and SST in the western Arabian sea (ρ = -0.71 for SPI and -0.8 for PDSI) (b-c). Apparently, the variability in WTIO has contributed to severe droughts in NRB.


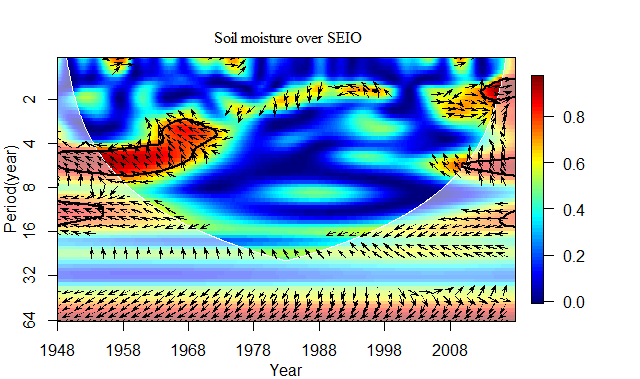

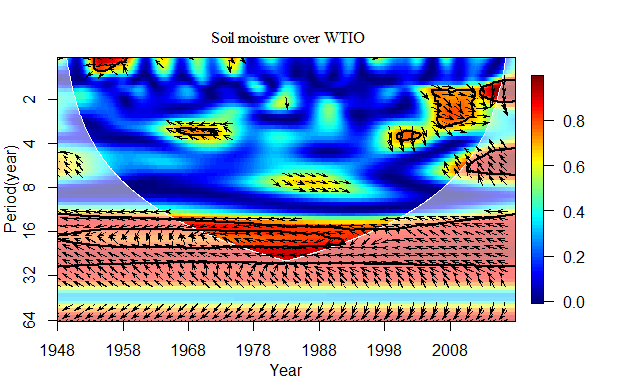


**c**

**b**

**a**


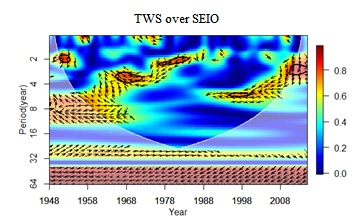

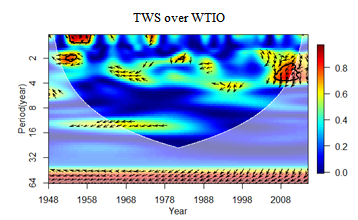


**d**

**Supplementary Fig. 14:** Influence of WTIO and SEIO on NRB’s SMC and TWS. WTC between SMC and SEIO and WTIO (a, b), WTC between TWS and SEIO and WTIO (c, d). SMC and TWS were computed same as in supplementary Fig. 3 and then correlated with WTIO and SEIO amplitudes. In contrast to the in-phase relationship between IOD and SMC and TWS over 1948-2017, there are stronger anti-phase relationships at 16-32- and 32-64-year bands between WTIO (SEIO) and soil moisture (TWS) (a-d). There is also a significant negative correlation between NRB SMC with SEIO and WTIO amplitudes (ρ = -0.83 for WTIO and -0.92 for SEIO). This strong negative correlation indicates that the reduction in the NRB SMC is strongly linked to increased SEIO and WTIO amplitudes. In f, the strong negative correlation between TWS and SEIO and WTIO (ρ = -0.91 for WTIO and -0.95 for SEIO) implies that the large deviations in the Indian ocean SST gradient over the Arabian Sea and southeastern parts of the Indian Ocean are the main controllers of the NRB’s TWS. This result suggests that the Arabian Sea (western pole) and the eastern Indian Ocean SST (southeastern pole) explains more of the variability of NRB’s SMC and TWS than IOD, for both SMC and TWS of NRB were positively correlated with IOD amplitude.


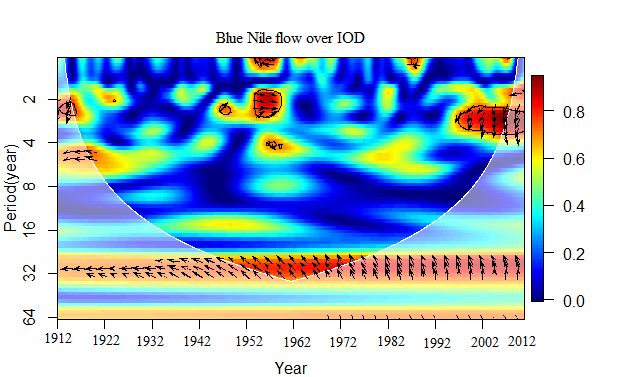


**a**


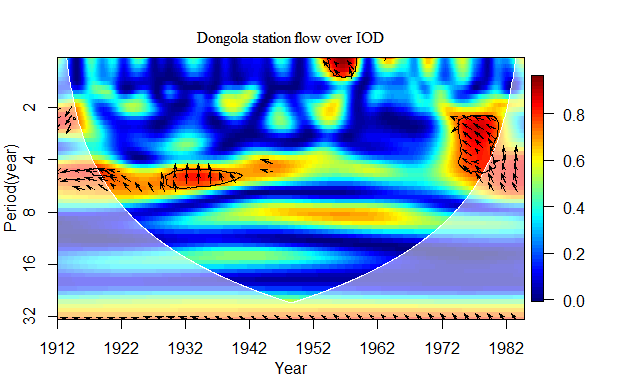


**b**


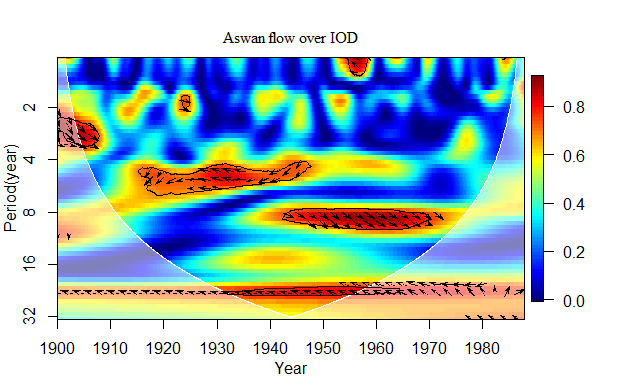


**c**

**Supplementary Fig. 15.** WTC between observed monthly flows and IOD: (a) IOD and Blue Nile flow, (b) IOD and Dongola flow, and (c) IOD and Aswan station flow. The WTC between the Blue Nile flow and IOD shows a strong anti-phase relationship at 32-year band over 1905-2012 and a stronger anti-phase relationship at 2-4- and 4-8-year bands over 2000-2012. In b, the Nile flow at Dongala station and IOD also exhibited significant anti-phase relationship after 1970s at 2-4 and 4-8 year while the Nile flow at the Aswan station (downstream) showed anti-phase relationships with IOD at 20-25 year band over 1930-1984 (c).

**Supplementary** Table 5: Nile River flow trend change detection and trend analysis:

|  |  | **Pettitt test** | | | | **Mann–Kendall test** | | | |  |  |
| --- | --- | --- | --- | --- | --- | --- | --- | --- | --- | --- | --- |
| **Nile river basin** | Years | K | T | P | trend | Tau | Sen's slope | P | trend | Mb | Ma |
| Monthly flow “Aswan dam” | 1900-1987 | 40862 | 1965 | 0.001 | Ha | 0.134 | 3.966 | <0.0001 | - | 2637 | 2016 |
| Annual flow “Aswan dam” | 1900-1987 | 1446 | 1965 | <0.0001 | Ha | 0.2063 | 11.41 | 0.027 | - | 2754 | 2247 |
| Flow anomaly “Aswan dam” | 1900-1987 | 1446 | 1965 | <0.0001 | Ha | 0.2063 | 11.41 | 0.027 | - | 249.7 | -716.5 |
| Monthly flow “Dongola” Sudan | 1910-1984 | 15669 | 1945 | 0.02 | Ha | 0.010 | 2.008 | 0.05 | - | 2687 | 2567 |
| Annual flow “Dongola” Sudan | 1910-1984 | 658 | 1965 | 0.001 | Ha | 0.278 | 7.74 | 0.008 | - | 2754 | 2247 |
| Flow anomaly “Dongola” Sudan | 1910-1984 | 658 | 1965 | 0.001 | Ha | 0.241 | 7.74 | 0.003 | - | 132.05 | -375.3 |
| Monthly flow “Blue Nile” Sudan | 1912-2010 | 17702 | N | 0.6 | Ha | 0.04 | 0.61 | 0.044 | - | 1575 | |
| Annual flow “Blue Nile” Sudan | 1912-2010 | 854 | 1964 | 0.015 | Ha | 0.08 | 1.37 | 0.15 | - | 1645 | 1478 |
| Flow anomaly “Blue Nile” Sudan | 1912-2010 | 854 | 1964 | 0.015 | Ha | 0.054 | 1.37 | 0.14 | - | 77.68 | -89.5 |

**Supplementary** Fig 16. Historical CMIP5 and future projection of warming trend (a), precipitation (b), potential evapotranspiration (c), soil moisture content (d), relative humidity under three climate change scenarios (e), and projected change in El Niño 3.4 indix (f).

**Supplementary** Table 6: Cross-correlation coefficients and standard error between El Niño 3.4, IOD, WIO, SEIO and NRB’s climate, drought indices and streamflow

|  | El Niño 3.4 | | IOD | |
| --- | --- | --- | --- | --- |
| **Variable** | **ρ** | **error** | **ρ** | **error** |
| Ts | 0.97 | 0.042 | 0.35 | 0.042 |
| GPH | 0.81 | 0.042 | -0.57 | 0.042 |
| precipitation | -0.7 | 0.042 | -0.37 | 0.042 |
| RH | -0.97 | 0.042 | 0.59 | 0.042 |
| AET | 0.93 | 0.042 | -0.47 | 0.042 |
| Meridional wind | 0.8 | 0.042 | -0.38 | 0.042 |
| Zonal wind | -0.11 | 0.042 | 0.045 | 0.042 |
| SPI | -0.86 | 0.042 | -0.87 | 0.042 |
| SPEI | -0.89 | 0.042 | -0.25 | 0.042 |
| sc-PDSI | -0.75 | 0.042 | -0.41 | 0.042 |
| Runoff | -0.84 | 0.042 | -0.1 | 0.042 |
| Dongala flow | -0.31 | 0.042 | -0.71 | 0.042 |
| Blue Nile flow | 0.25 | 0.042 | -0.57 | 0.042 |
| SMC | -0.72 | 0.042 | 0.43 | 0.042 |
| TWS | -0.61 | 0.042 | 0.52 | 0.042 |

**Supplementary** Table 7: Cross-correlation coefficients and standard error between WIO, SEIO and NRB’s climate, drought indices and streamflow

| **Variable** | WIO | | SEIO | |
| --- | --- | --- | --- | --- |
|  | **ρ** | **error** | **ρ** | **error** |
| precipitation | -0.82 | 0.035 | -0.36 | 0.035 |
| SPI | -0.71 | 0.035 | -0.24 | 0.035 |
| SPEI | -0.67 | 0.035 | -0.16 | 0.035 |
| sc-PDSI | -0.8 | 0.035 | -0.32 | 0.035 |
| Runoff | -0.41 | 0.035 | -0.138 | 0.035 |
| Dongala flow | -0.72 | 0.035 | -0.58 | 0.035 |
| Blue Nile flow | -0.92 | 0.035 | -0.88 | 0.035 |
| SMC | -0.83 | 0.035 | -0.92 | 0.035 |
| TWS | -0.91 | 0.035 | -0.95 | 0.035 |
